# Volume electron microscopy

**DOI:** 10.1038/s43586-022-00131-9

**Published:** 2022-07-07

**Authors:** Christopher J. Peddie, Christel Genoud, Anna Kreshuk, Kimberly Meechan, Kristina D. Micheva, Kedar Narayan, Constantin Pape, Robert G. Parton, Nicole L. Schieber, Yannick Schwab, Benjamin Titze, Paul Verkade, Aubrey Aubrey, Lucy M. Collinson

**Affiliations:** 1 Electron Microscopy Science Technology Platform, The Francis Crick Institute, London, UK; 2 Electron Microscopy Facility, Faculty of Biology and Medicine, University of Lausanne, Lausanne, Switzerland; 3 Cell Biology and Biophysics Unit, European Molecular Biology Laboratory, Heidelberg, Germany; 4 Department of Molecular and Cellular Physiology, Stanford University, Palo Alto, CA, USA; 5 Center for Molecular Microscopy, Center for Cancer Research, National Cancer Institute, National Institutes of Health, Bethesda, MD, USA; 6 Cancer Research Technology Program, Frederick National Laboratory for Cancer Research, Frederick, MD, USA; 7 The Institute for Molecular Bioscience, The University of Queensland, Brisbane, Queensland, Australia; 8 Centre for Microscopy and Microanalysis, The University of Queensland, Brisbane, Queensland, Australia; 9 Cell Biology and Biophysics Unit/ Electron Microscopy Core Facility, European Molecular Biology Laboratory, Heidelberg, Germany; 10 Nanosurf AG, Liestal, Switzerland; 11 School of Biochemistry, University of Bristol, Bristol, UK; 12 Janelia Research Campus, Howard Hughes Medical Institute, Ashburn, VA, USA; 13 Present address: Faculty of Biosciences, Heidelberg University, Heidelberg, Germany

## Abstract

Life exists in three dimensions, but until the turn of the century most electron microscopy methods provided only 2D image data. Recently, electron microscopy techniques capable of delving deep into the structure of cells and tissues have emerged, collectively called volume electron microscopy (vEM). Developments in vEM have been dubbed a quiet revolution as the field evolved from established transmission and scanning electron microscopy techniques, so early publications largely focused on the bioscience applications rather than the underlying technological breakthroughs. However, with an explosion in the uptake of vEM across the biosciences and fast-paced advances in volume, resolution, throughput and ease of use, it is timely to introduce the field to new audiences. In this Primer, we introduce the different vEM imaging modalities, the specialized sample processing and image analysis pipelines that accompany each modality and the types of information revealed in the data. We showcase key applications in the biosciences where vEM has helped make breakthrough discoveries and consider limitations and future directions. We aim to show new users how vEM can support discovery science in their own research fields and inspire broader uptake of the technology, finally allowing its full adoption into mainstream biological imaging.

## Introduction

Volume electron microscopy (vEM; also referred to as volume EM) describes a set of high-resolution imaging techniques used in biomedical research to reveal the 3D structure of cells, tissues and small model organisms at nanometre resolution. vEM techniques leverage the unique ability of electron microscopes to reveal the structure of cells and tissues with the contrast and resolution to distinguish cell membranes and membrane bilayers. In the context of this Primer, and to delineate boundaries with other fields, we consider vEM to include any method of generating serial images in an electron microscope from resin-embedded cells and tissues with a continuous depth greater than 1 μm. This includes techniques based on transmission electron microscopy (TEM), in which the electron beam passes through an ultra-thin slice of the sample to generate an image, and scanning electron microscopy (SEM), in which the electron beam is raster-scanned over the surface of a sample generating an image from secondary and/or backscattered electrons. TEM-based vEM techniques such as serial section TEM (ssTEM) and serial section electron tomography (ssET) and SEM-based vEM techniques such as array tomography, serial blockface SEM (SBF-SEM; also known as SBEM) and focused ion beam SEM (FIB-SEM) all generate a series of images that can be stacked to form a 3D digital representation of the original sample volume. Further, we cover emerging vEM technologies such as automated serial TEM (TEMCA and GridTape TEM), fast ion beam technologies (plasma and gas cluster ion beam systems) and massively parallel imaging in SEM (multibeam SEM (mSEM) and FAST-EM).

vEM techniques have emerged over the past 20 years, largely in response to the demands of the connectomics field ^1^. Connectomics has the dual challenge of imaging neurons in the brain with sufficient volume to capture their length and with sufficient resolution to image individual synapses, as structural indicators of functional connections between cells. The development of vEM was enabled by key technological advances in sample preparation, in automation of sample sectioning and TEM and SEM sources and detectors and, importantly, in scientific computing to facilitate acquisition and analysis of large image datasets. The biomedical applications of vEM quickly moved beyond connectomics, as the value of imaging complex biological processes in 3D in tissues and model organisms became clear. vEM is now widely used in studies of cell structure and non-brain tissue in fields as diverse as infection, immunity, cancer research, neurodegeneration, developmental biology, plant biology, synthetic biology, biomechanics, biomaterials and clinical research.

Scientists who develop and use vEM technology come from a wide variety of research disciplines. At first glance, it would seem to be difficult to form a coherent community from such a diverse group. However, over the past decade, grassroots initiatives have gathered technologists, microscopists, computational scientists, biologists and clinical scientists together to drive the development and application of vEM. The European Molecular Biology Organization (EMBO) and microscope manufacturers have been particularly active in supporting the organization of workshops and symposia ^2^, and more recently the community has self-organized around a set of working groups to collaborate globally on vEM research, development and application (see the volume EM community website).

In this Primer, we present the state of the art and future perspectives for new audiences who wish to leverage the power of vEM in their own research. We outline the principles and practical steps required to move from a living biological entity to an image volume representing the sample, covering sample preparation and imaging using a range of vEM modalities (Experimentation), consider how to handle and analyse the massive amounts of image data produced by vEM (Results) and illustrate how vEM is used in real-world research, revealing structure and function across scales from organelles to organisms (Applications). Limits to the throughput of vEM and the importance of sharing rich vEM datasets (Reproducibility and data deposition) and how bottlenecks might be overcome (Limitations and optimizations) are discussed. Finally, we consider the challenges and opportunities of vEM for mining new understanding from complex multiscale biological systems (Outlook). Although we do not include electron microscopy techniques that generate serial images from frozen hydrated samples, such as cryo electron tomography or serial imaging in the cryo FIB-SEM, we address the exciting and challenging crossover between the worlds of vEM and cryo electron microscopy (cryo-EM) in the final section of the primer.

## Experimentation

**Figure 1:**
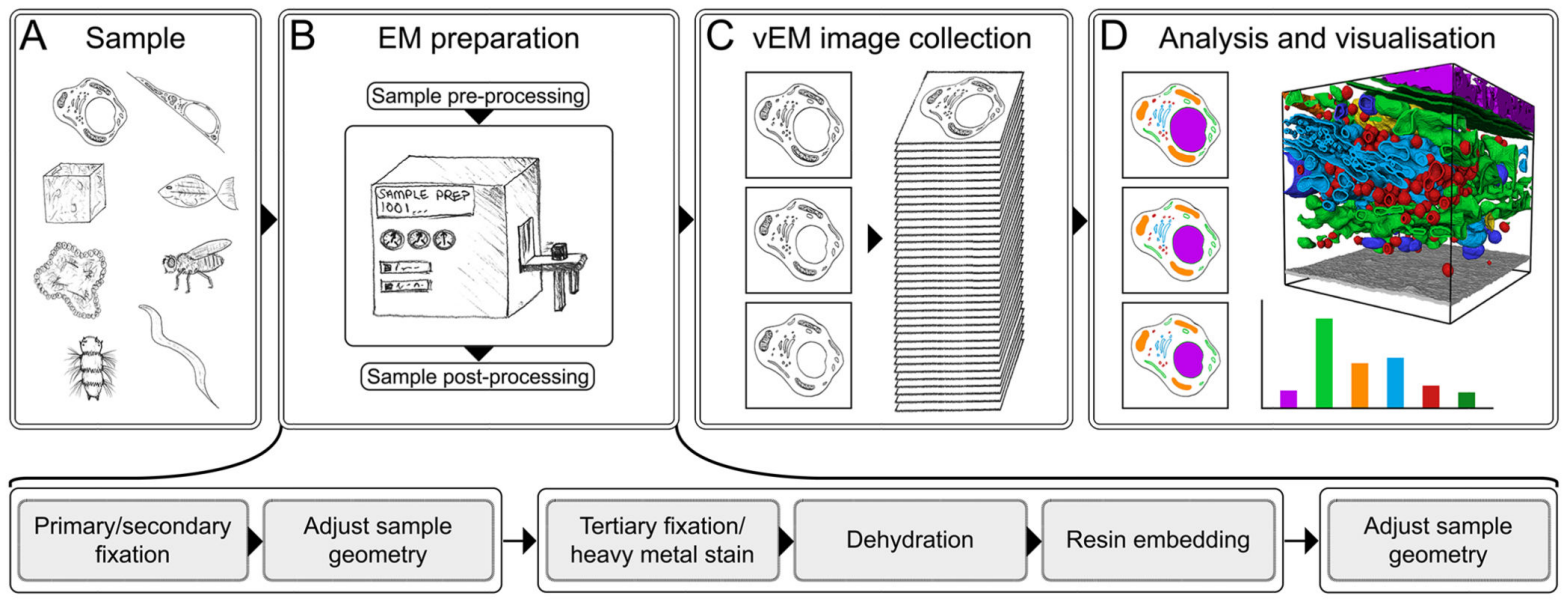
A typical vEM workflow. Properties of the sample, the target structure and research question determine the overall design of a volume electron microscopy (vEM) workflow. **A:** A wide variety of sample types can be examined, including isolated cells and cell monolayers, individual model organisms (including *Danio rerio, Drosophila melanogaster, Caenorhabditis elegans* and *Platynereis* larvae) and model systems such as organoids and tissues. **B:** The sample undergoes numerous complex preparation steps to produce a specimen optimized for vEM imaging. The sample must first be preserved in as near a native state as possible using chemical or cryogenic fixation methods. In correlative vEM workflows, light microscopy is typically carried out before processing for vEM, either pre fixation or post fixation. The geometry of large samples must be modified to make them compatible with subsequent staining steps owing to limited penetration of reagents. The sample is then exposed to application-specific cocktails of heavy metals, dehydrated and embedded in resin to both introduce electron contrast to the features of interest, and stabilize the sample within the vacuum of the microscope. Once embedded, the samples then undergo a further process of application-specific geometry modification to meet constraints of the target imaging modality. **C:** All vEM modalities result in a stack of serial images. **D:** The image volume is then reconstructed, analysed and visualized using a varied mixture of manual, semi-automated and automated algorithms in opensource and commercial software. Imaged volume in panel D adapted from Heinrich et al. ^158^.

A vEM experiment encompasses multiple steps (Fig.1), each of which must be carefully planned to maximize the chances of a successful outcome (Box 1). Steps to be decided on and optimized include the choice of biological sample and experimental conditions, considering that the number of replicates is limited by the complexity and throughput of the technique; the probes that will be used to mark the region of interest (ROI) within the sample and add molecular information to the ultrastructure; the sample preparation protocol(s) that will be used to preserve the sample as close to living state as possible whilst enhancing compatibility with electron imaging in a vacuum; the strategy for tracking and trimming the sample to the ROI for imaging; the choice of vEM imaging modality, considering the desired field of view and resolution of the image data; and the size and complexity of the data analysis task with respect to producing a useful biological interpretation of the results.

### Sample preparation

In this primer, we refer to the initial biological material as the sample, and the prepared material that is ready for imaging as the specimen (Fig.1). Almost any biological sample can be imaged using vEM, from prokaryotic cells through to eukaryotic cells, tissues and model organisms, although the volume and resolution dictates how much of a sample can be imaged. vEM of biological samples is underpinned by extensive optimization of sample preparation ^3^. A successful experiment relies on protocols that ensure the sample is kept as close as possible to its native state but also in a form that can be imaged ^4^. The specimen must be able to withstand the vacuum inside the electron microscope, as well as the high energy and current of the electron and/or ion beam; to deliver sufficient image contrast; and to exhibit sufficient structural rigidity, especially for volume slicing ^5^. Protocols are often tweaked to account for the properties of each sample, target structure and imaging modality, and thus are highly variable within the community. We give a generalised overview of sample preparation below, covering optimization of the sample size, fixing the sample, staining with contrast dyes, dehydrating the sample and embedding it into a resin, and trimming the resin block.

#### Optimization of sample size

The maximum sample size is dictated by the depth of penetration of fixatives and stains. It is generally recommended that the sample is less than 1 mm thick in at least one dimension, although the use of customised microwave ovens to increase the permeability of samples and the diffusion of solutions is pushing this limit ^6^. Cell monolayers present no issues, with a thickness of around ten microns when grown on glass or plastic, and cell suspensions can be centrifuged to a pellet of suitable size ^7,8^. Model organisms in the millimetre range, like *Caenorhabditis elegans* ^9,10^, *Drosophila melanogaster* ^11^ and *Danio rerio* ^12,13^, may be dissected or imaged in their entirety. Tissue in the centimetre range is usually dissected into smaller pieces or cut into slices using a vibratome ^14^, although mouse brain has been successfully processed intact for connectomics ^15^.

#### Fixation

Two avenues can be taken for fixation, depending on the sensitivity of the biological structure under investigation and the size of the sample. Chemical fixation using combinations of methanol-free (para)formaldehyde (2–4%) and glutaraldehyde (1–4%) is straightforward; however, this may not capture fast subcellular events such as membrane tubulation ^16^. Alternatively, cryo-fixation using high pressure freezing (combined with freeze substitution to transition the sample to resin) captures fast biological events occurring at the scale of milliseconds and minimizes shrinkage artefacts, although this method requires complex equipment ^17–19^. The choice between chemical fixation and cryo-fixation is mostly determined by the initial size of the sample; high-pressure freezing is limited to samples with dimensions of approximately 200 μm, whereas millimetre-sized samples can be fixed through perfusion of chemical fixatives. As a result, cell monolayers, cell suspensions, *C.elegans* and *D.melanogaster* are particularly well suited to high-pressure freezing owing to their small size. Further detailed guidance on fixation choice is available elsewhere ^3–4,14,17,19^.

#### Contrasting

The addition of staining to enhance electron contrast of biological samples is a critical step in vEM to improve the visibility of membranes and make the sample conductive. The vEM imaging modality chosen influences the level of staining required: TEM-based techniques are compatible with lower contrast levels, partially owing to an improvement in detectors and because images are generated by scattering of primary electrons; ion beam techniques require medium levels of contrast because images are formed by backscattered electrons; and in situ microtome techniques require high levels of staining to reduce the beam dose required to generate a backscattered electron image to prevent charging artefacts and resin de-polymerization. Protocols used by the community are highly variable, but most involve immersion of the sample into solutions containing combinations of heavy metal salts to introduce contrast, including uranium, lanthanides ^20^, osmium (reduced or aqueous), thiocarbohydrazide, tannic acid ^21^ and pyrogallol ^15,22^. If an experiment can tolerate light staining, then uranium salts only may be used ^23^, but most samples require heavy staining with layers of reduced osmium, thiocarbohydrazide and osmium, followed by uranium salts and lead aspartate ^24–27^. These modern protocol variations are all based on historical work ^28–30^, with new variations optimized for specific sample types appearing regularly in the literature ^6,15,22,31^. Furthermore, the use of uranium is prohibited in several countries, leading to the development of replacement compounds ^32,33^.

#### Dehydration and resin embedding

To protect samples from the electron microscope vacuum and facilitate slicing of the sample into ultra-thin sections, water in the cells and tissues is removed by dehydration with a solvent that is compatible with subsequent liquid resin infiltration, which is then polymerised to form a hard block. Chemically fixed samples are dehydrated using a graded series of ethanol or acetone, whereas high pressure frozen samples are contrasted and dehydrated simultaneously during a process called freeze substitution ^23,34,35^. Following dehydration, a graded series of resins are used to infiltrate the sample before it is hardened using heat ^36,37^ or UV light ^38,39^. Epoxy resins such as Durcupan ^40^, Spurrs ^41^, Epon 812 and Hard Plus are most commonly used due to their good sample penetration properties and stability during imaging. Methacrylate resins such as Lowicryl HM20 and LR White may also be used, particularly when preserving the emission of fluorescent proteins in the sample.

#### Trimming

To prepare the sample for ultra-thin sectioning or milling, and to optimize its volume to the downstream imaging modality being used, the sample is manually trimmed using a razor blade or diamond knife in an ultramicrotome. Many samples require prior knowledge of the position of the target structure to guide trimming, which is usually achieved by integrating fluorescence microscopy and/or X-ray microscopy (Box 2) into a correlative workflow in order to locate and follow the target structure during trimming ^23,35,42-46^. Once trimmed, specimens may be mounted directly for vEM, or cut into ultra-thin sections using a diamond knife, depending on the downstream imaging modality. Removal of excess resin before polymerization ^47–49^, or use of a conductive coating ^50^ can minimize trimming and facilitate targeting of biological structures by revealing surface anatomy. Large resin-embedded tissue samples may also be subdivided using a hot knife for vEM of serial slabs ^51,52^.

### Probes

vEM techniques provide an opportunity to correlate morphological data with molecular characterization. The ideal labelling method for vEM should enable molecular localization throughout the depth of the sample, providing molecular resolution with the sensitivity to detect molecules at endogenous levels in their native non-perturbed environment. This is challenging but has been achieved through the development and application of a suite of genetically encoded tags.

Genetically encoded tags that produce electron-dense reaction products that are visible in vEM have been described, including metallothionein-based probes ^53–56^, reASH ^57^, miniSOG ^58^ and APEX/APEX2 ^59^. Of these, APEX2 (an evolution of the original APEX probe that has higher activity in cells) is the most established tag. It is an ascorbate peroxidase that is active in both cytosolic and luminal/extracellular compartments, and is proving to be a robust method with a large number of applications including detection of directly tagged proteins of interest ^60^, nanobody-directed detection of GFP and RFP-tagged proteins ^61^ and labelling of specific protein complexes using a split GFP-based method ^62^ or split-APEX ^63^. APEX converts soluble diaminobenzidine (DAB) into an insoluble electron-dense reaction product ^59^. APEX can be used with chemically fixed specimens or in cryo-EM schemes (cryoAPEX) ^64^ and has widespread applicability in both cell and tissue/animal systems, even enabling multiplexed labelling of different cell populations in tissue ^65^. The APEX-directed DAB reaction product can also nucleate the formation of gold colloids ^66,67^, a method compatible with single-molecule detection as shown using genome-edited cells with tagged proteins at endogenous levels ^67^. Single molecule detection is also possible using an elegant technique based on a metallothionein tag that can be used to nucleate gold particles and, potentially, could have applications in different vEM modalities ^68^.

Fluorescent genetically encoded tags can also be used to locate structures in vEM, either indirectly or directly, using a range of correlative light and volume electron microscopy (vCLEM) workflows. In addition to genetically encoded tags, antibodies and neural tracers have been used to label organelles and cells for vEM. The recent development of a method for immunolabeling throughout larger volumes of tissue (sections up to 1 mm thick), without permeabilization, retains good ultrastructure and allows the collection of molecular information using vCLEM with SBF-SEM ^69^. Array tomography ^70,71^ allows labelling on sections, removing the limitation of depth penetration into hydrated tissue samples, but is dependent on the availability of electron microscopy-compatible antibodies and epitope preservation during sample preparation. Using a combination of neural tracers ^72^, expressed fluorescently-tagged proteins and on-section immunolabelling opens the possibility of multiplexed labelling in electron microscopy. Another strategy for multiplexed labelling with ssTEM is to pull out a few ultra-thin sections from the series, stain them using immunohistochemistry ^73^ or immunogold labelling ^74^ and, then, propagate the molecular information through the serial image stack.

### TEM-based vEM

**Figure 2:**
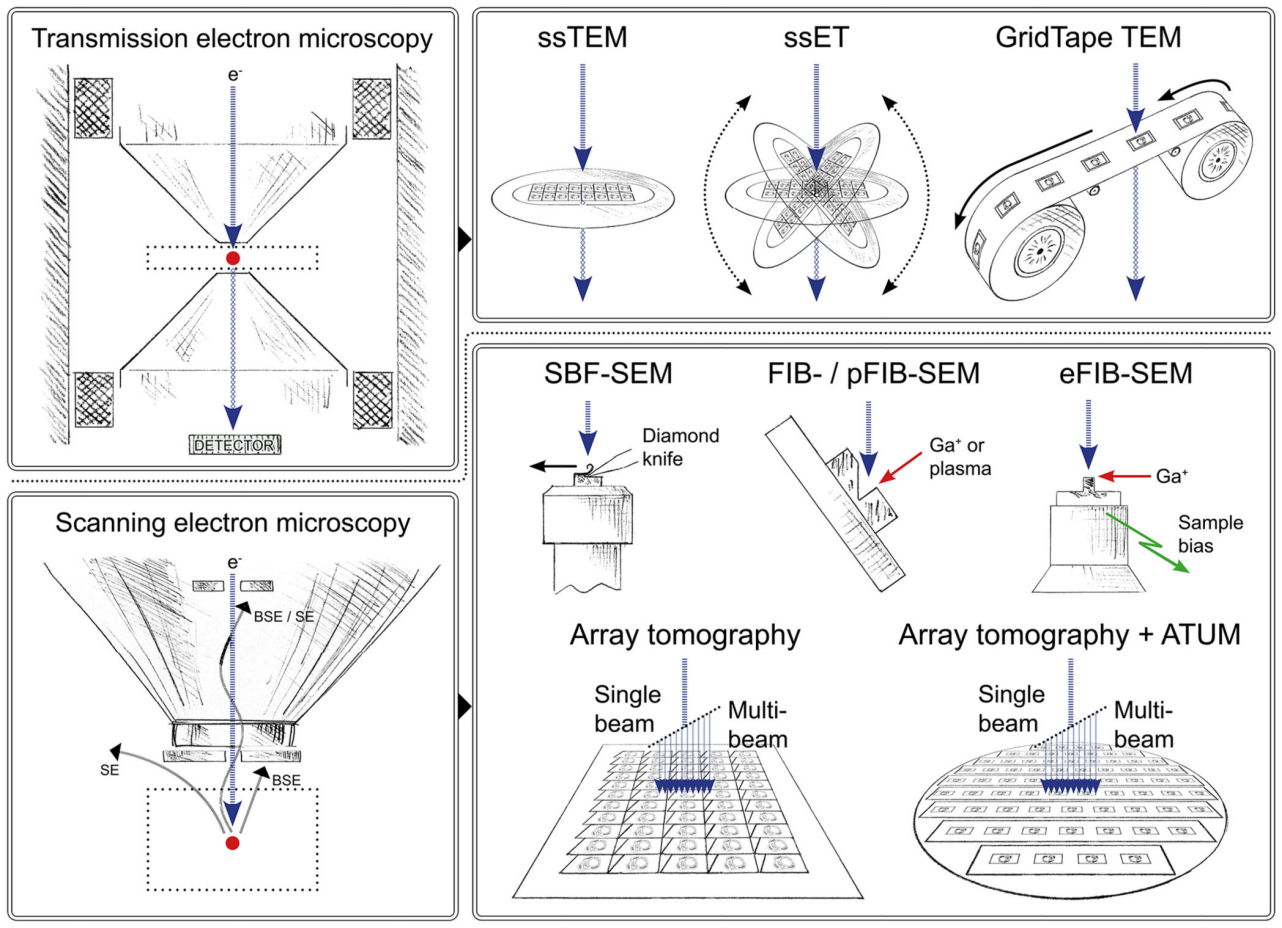
vEM encompasses a collection of closely related imaging modalities. Volume electron microscopy (vEM) is a collective term for numerous imaging modalities based on transmission electron microscopy (TEM) and scanning electron microscopy (SEM). Within each modality, overall sample position (dotted box, with a red spot indicating the location of electron beam interaction) and signal generation mechanisms remain broadly similar. For TEM, electrons that pass through the sample are collected by a downstream detector. For SEM, backscattered (BSE) and/or secondary electrons (SE) are generated by electron beam interaction with the sample and recorded using in-chamber or incolumn detectors (pale blue). However, the format of sample and mechanism used for generating serial images vary. For TEM, serial ultra-thin sections of 50-70 nm thickness are typically collected on substrates coated with an electron transparent support film such as formvar-coated copper slot grids (serial section TEM (ssTEM)). 3-D volumes can also be generated by imaging through serial sections of 200-300 nm thickness at multiple tilt angles, back projecting resultant images and reconstructing the volume (serial section electron tomography (ssET)). Alternatively, very extensive collections of serial ultra-thin sections can be imaged using a tape-based support system that feeds directly through the electron column (GridTape TEM). For SEM, options range from sequentially cutting and imaging the sample using a miniaturized in-chamber ultramicrotome (serial blockface SEM (SBF-SEM)) or directly milling from the imaged surface using a gallium ion beam (focused ion beam SEM (FIB-SEM)) or a variety of plasma moieties (pFIB-SEM). Enhancements such as sample bias, modified sample geometry and closed-circuit milling control are integral to some advanced workflows (enhanced FIB-SEM (eFIB-SEM)). Imaging of serial ultra-thin sections collected on a suitable substrate such as silicon wafer or indium tin oxide-coated glass (array tomography), or Kapton tape (array tomography with automated tape-collecting ultramicrotome (ATUM)) can also be achieved using SEM, with either a single electron beam, or parallel electron beamlets.

Volume imaging using TEM (Fig.2) combines the benefits of high resolution, non-destructive imaging with the speed of full-field (as opposed to scanning) acquisition using CCD or CMOS cameras. The geometry of the transmission electron microscope limits the size and thickness of the specimen that can be imaged so an ultramicrotome with a diamond knife is used to first cut the sample into serial ultra-thin sections, which are picked up on support grids 3 mm in diameter. For ssTEM, section thickness is generally between 50 and 100 nm. For ssET, slightly thicker sections of 200 to 300 nm are imaged, and resolution through the volume is increased by collecting a series of images at different tilt angles (usually ±70°), which are then reconstructed into a digital volume. To improve the reconstruction, dual tilts may be collected at 90° to each other. In both ssTEM and ssET, serial sections are imaged sequentially and the resulting images stacked to represent the sample volume.

ssTEM delivered the first of the whole model organism electron microscopy volumes in the Mind of the Worm, a map of the neural connectivity of *C.elegans* ^75^. ssET has also delivered insights into the complexity of subcellular structures ^76,77^. However, these techniques are slow, low throughput, manual and technically challenging, severely limiting the volumes that can be imaged. Efforts to address these limitations in TEM-based vEM have focused on solving the dual challenges of the limited field of view imposed by commercial detectors and automation of section retrieval onto electron-transparent supports for imaging in transmission mode.

One of the first computational solutions for large-scale ssTEM imaging enabled the automated acquisition and assembly of thousands of individual images from each section into a large mosaic, to acquire a terabyte volume of rabbit retina ^78^. On the instrumentation side, a fruitful approach was the development of TEMCA ^79^, which used a commercial 120 keV TEM customised with an array of detectors combined into a single large sensor. The microscope was supported on a raised platform to increase the distance between the sample and the detector, thereby increasing the field of view. However, although detection was optimized, these approaches still relied on manual collection of thousands of sections onto classical TEM slot grids. This issue was partially addressed using an autoloader on the TEM, with which grid exchange was automated using a robot synchronized with the imaging process ^80–82^. Grid handling was further improved by the development of GridTape TEM ^83,84^ (Supplementary Movie 1), in which sections were cut using a modified ultramicrotome for collection on a tape with electron transparent film-covered holes. The tape was fed through a modified sample holder on a customised transmission electron microscope, and each section automatically imaged using a fast array of detectors, enabling the motor control circuits in adult *D. melanogaster* to be mapped ^84^. In parallel, a second system called piTEAM has been developed, consisting of a parallelized array of transmission electron microscopes optimized for GridTape imaging ^85^. The speed, automation and non-destructive nature of GridTape TEM has the potential to transform peta-scale vEM imaging while, simultaneously, creating new challenges in data handling and analysis.

### SEM-based vEM

Volume imaging using SEM (Fig.2) combines the benefits of large fields of view and a microscope chamber compatible with large specimen holders and in situ specimen slicing mechanisms. SEM-based vEM has been widely adopted and includes some of the most user-friendly vEM technology, allowing routine acquisition of large-volume image data with ultrastructural resolution ^5,86–89^. Three complementary but distinct approaches have been developed in parallel: those using in situ microtomes such as SBF-SEM; those using ion beams such as FIB, plasma FIB (pFIB) ^90^ or enhanced FIB-SEM (eFIB-SEM) ^52^, and array tomography ^5,70,87^. These three approaches all require heavily contrasted specimens; a scanning electron microscope optimized for low voltage imaging (1-2 keV) with a field emission gun and backscattered or secondary electron detectors; and dedicated software and a stable system for autonomous imaging over days, weeks or even months.

#### In situ microtomes

There are currently three commercial SBF-SEM (Fig.2 and Supplementary Movie 2) products available, to our knowledge - 3View (Gatan Inc., now part of Ametek Inc.), VolumeScope (ThermoFisher Scientific Inc.) and Katana (ConnectomX Ltd) - as well as several home-made solutions ^91^. All rely on the same principle - a diamond knife held in a miniaturised ultramicrotome mounted in the SEM chamber moves back and forth across the resin block, with the block moving upwards in z in incremental steps that determine the section thickness ^92,93^. In all solutions, image tiling is possible by moving the entire microtome in the chamber. This technique has the advantage of imaging large volumes ^50,91^ but the disadvantage of suboptimal working distance, restricting resolution and choice of detector. Charging artefacts caused by poor conductivity of the sample blocks are a common problem ^94^. The first versions of in situ microtomes relied on variable pressure in SEM to mitigate charging ^92^, which is a particular problem for specimens that contain large areas of non-conductive empty resin such as cell pellets or lung tissue. Now, newer instruments use a focal charge compensation system ^95^ to inject a gas flow locally at the level of the blockface to mitigate charging, increasing the range of specimens that can be imaged.

#### Ion beams

Alongside the development of in situ microtomes, researchers and manufacturers explored the usage of a gallium FIB to remove thin sections from resin blocks inside a scanning electron microscope ^96–98^ (Fig.2 and Supplementary Movie 2) By replacing mechanical sectioning with electronically controlled ion milling, this technique has the advantage of shaving thinner slices than in situ microtomes (typically 50 nm for in situ microtomes versus 5 nm for FIB-SEM) and achieves higher resolution with a better signal-to-noise ratio ^52^. The possibility of obtaining isotropic voxels, where the slice thickness in z matches the pixel size in XY, makes FIB-SEM attractive for automatic segmentation ^99^. Uniquely, the direction of ion beam milling is perpendicular to that of other sectioning techniques, an important point to consider during experimental design, to ensure that the region to be imaged is accessible to the electron beam ^100^. However, the ion beam loses energy as it progressively mills down the blockface, limiting the depth that can be precisely cut ^52^. In order to improve speed and throughput, specimens have been imaged in parallel on multiple eFIB-SEM instruments that are more stable and resilient to errors over imaging runs of many months ^52^. As negative charges imparted to the imaged surface by the electron beam are mitigated by subsequent exposure to positive ions from the milling beam (and concomitant removal of this charged slice), FIB-SEM can be used to image poorly contrasted specimens in which the emission of fluorescent proteins has been preserved ^23^. Recently, pFIB instruments with other ion species have been used for milling biological specimens, including oxygen ^90^, xenon ^101^ and argon ^102,103^, which may offer finer or faster milling than gallium. Newer instruments use oxygen plasma ^104^, gas cluster ion beams ^105^ and multi-ion species to increase the milling speed and surface area.

#### Array tomography

Whereas in situ microtomes and ion beams both work by iterative imaging of the blockface, array tomography is based on SEM imaging of pre-cut ultra-thin serial sections arranged in two dimensions on a solid substrate (Fig.2 and Supplementary Movie 1). Serial sections are often obtained using a standard ultramicrotome and collected on either silicon wafers ^106,107^, or carbon-coated ^108^ or indium tin oxide-coated glass coverslips ^107,109^. Manual section pickup is challenging, requiring advanced expertise and manual dexterity, and is commonly used for smaller numbers of serial sections (< 500) ^110^. To overcome this limitation, various automated or semi-automated methods have been developed ^71^, including the Artos 3D (Leica Microsystems), the ASH-100 ^107^ and ASH2 (RMC Boeckeler), fishing ^111^, MagC ^112^, a modified knife ^106^, AutoCUTS SEM ^113^ and the Arraybot ^71^. An alternative and reliable approach for producing large numbers of serial sections is the automated tape-collecting ultramicrotome (ATUM) ^114^. This system, as the name implies, collects the serial sections on tape, which is then mounted on wafers for imaging. Commercial solutions for array tomography are becoming more widely available, mainly facilitated by advances in software automation for serial imaging. For imaging large volumes, mSEM and FAST-EM offer fast imaging at nanometre resolution by splitting the electron beam into multiple beamlets that image the sample in parallel ^115–117^. Compared to in situ microtomes and ion beams, array tomography has a lower z-resolution (a minimum of 30 nm). However, because this approach preserves the sections, it has the advantage that the specimen can be re-imaged in different areas or at different magnifications. Furthermore, the sections can be imaged at the light level prior to electron microscopy, to collect molecular information from immunofluorescence, neural tracers or genetically encoded probes ^70,71,107,108^. Integrated light and scanning electron microscopes are a promising solution for efficient acquisition and registration of fluorescence microscopy and electron microscopy images from array tomography specimens ^109,118,119^.

### Multi-vEM workflows

**Figure 3:**
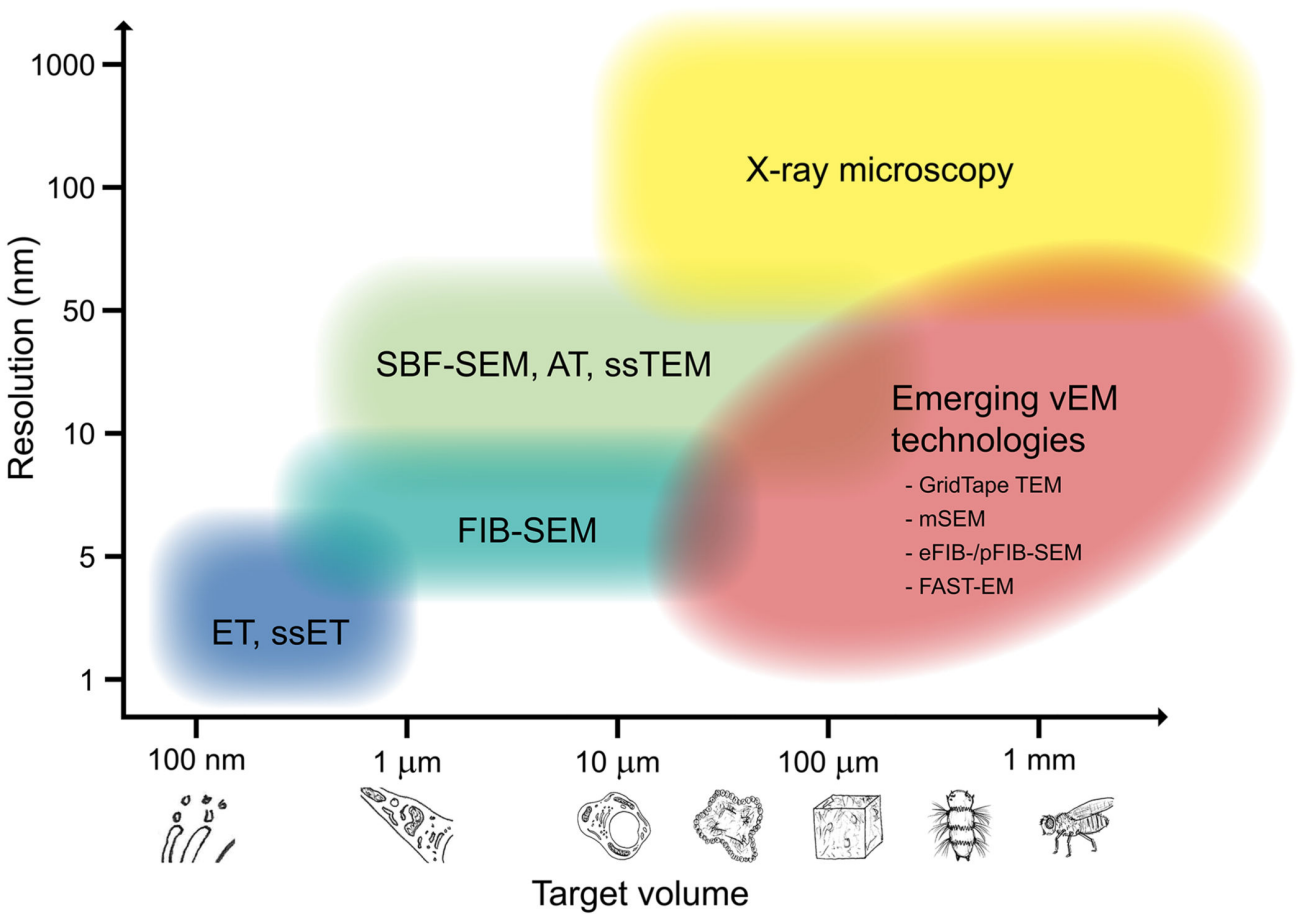
vEM as a multiscale multimodal imaging technique. Volume electron microscopy (vEM) continually pushes the boundaries of high-resolution volume imaging. Transmission electron microscopy (TEM)-based imaging modalities (serial section electron tomography (ssET) and serial section TEM (ssTEM)) tend to deliver higher resolutions, whereas scanning electron microscopy (SEM)-based modalities (serial blockface SEM (SBF-SEM), array tomography and focused ion beam SEM (FIB-SEM)) tend to deliver larger volumes. Multimodal workflows, emerging vEM technologies such as GridTape TEM, multibeam SEM (mSEM), enhanced FIB-SEM (eFIB-SEM), plasma FIB-SEM (pFIB-SEM) and FAST-EM, and associated techniques including X-ray microscopy are further extending scales and resolutions over which volume imaging is possible.

The research question, sample size and target feature may dictate a multiscale, multi-resolution workflow (Fig.3). In these circumstances, a multi-vEM approach is very powerful. For example, SBF-SEM can be used as a smart trimming tool to locate smaller regions for higher resolution imaging using FIB-SEM ^12,120^, TEM ^12^ or spatial compositional analysis using nanoSIMS ^121^. Other hybrid approaches have focused on the combination of thick serial sectioning and FIB-SEM to rapidly screen large volumes with acquisition of high-resolution isotropic information for targeted sub-volumes ^122,123^.

### vCLEM

**Figure 4:**
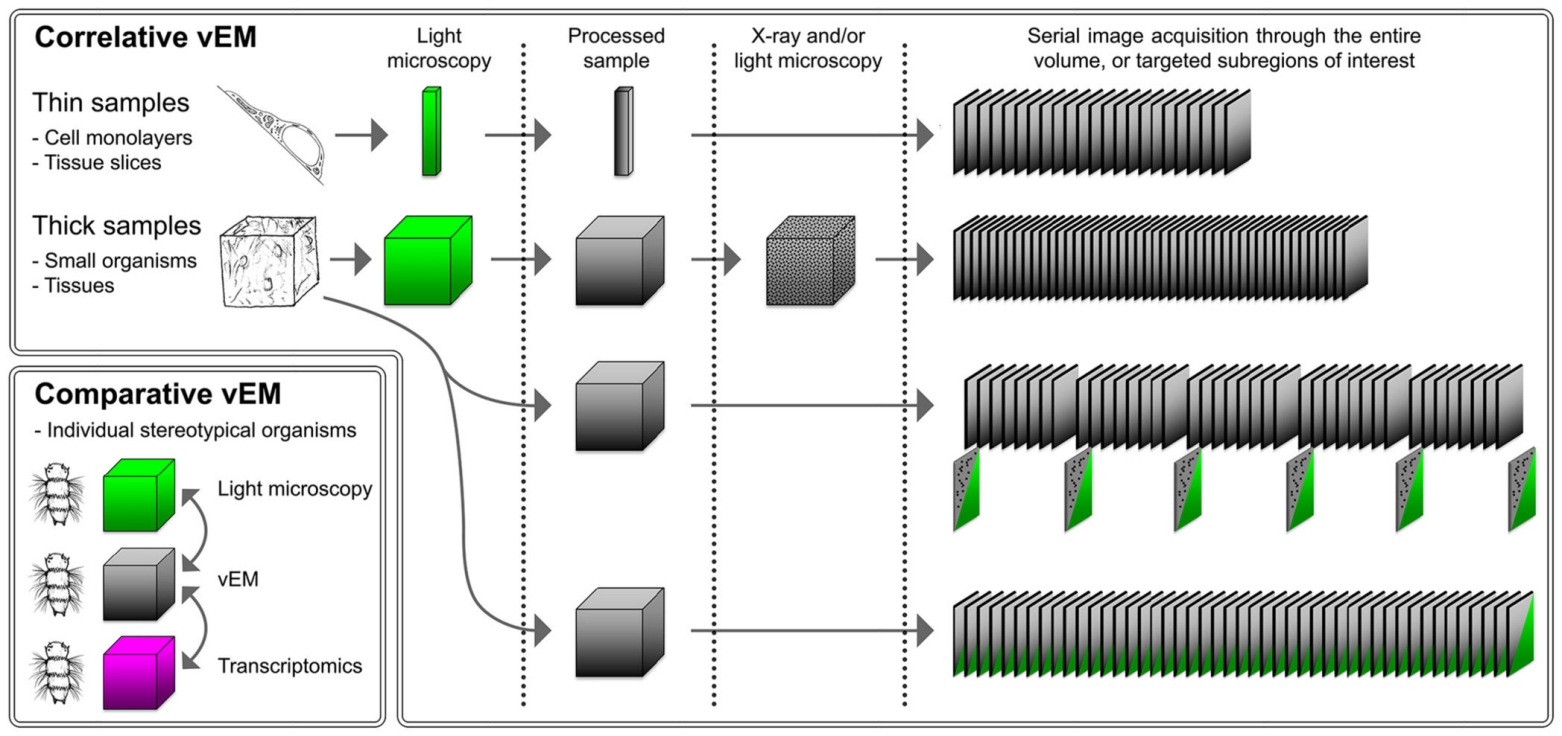
Correlative and comparative vEM for function-structure studies. Volume electron microscopy (vEM) studies frequently use multimodal imaging pipelines. Light microscopy, in particular, can be used to obtain a wide variety of molecular, developmental or functional information from a sample prior to processing for electron microscopy. Light microscopy can also be used to locate a region of interest (ROI) for targeted imaging (correlation for relocation) or to localize molecules to structures (correlation for registration). In thin samples, such as cell monolayers or tissue slices, the final volume is relatively small and correlation between light and electron imaging modalities is more straightforward. For successful correlation in thick samples, intermediate imaging steps are often required such as X-ray and light microscopy, in order to provide a link between low and high-resolution datasets and help to relocate the target area of interest prior to serial image acquisition throughout the target volume. Alternatively, intercalated serial section transmission electron microscopy (ssTEM) data sets can be probed by computational molecular phenotyping ^73^, or multiplexed siGOLD immunolabelling (black dots) ^74^, thereby providing a wealth of functional information that can be translated across the whole dataset. To further enhance a multiplexed volumetric approach, all sections can be used for each imaging modality, combining immunofluorescence and electron imaging modalities to link functional identification to the underlying tissue ultrastructure ^108^. Finally, for small stereotypical organisms such as *Platynereis* larvae, comparative analysis can be used to draw powerful conclusions for populations by correlating multimodal data from individual organisms ^50^.

Correlative microscopy techniques are an essential part of most vEM workflows, because the volume that can be imaged in electron microscopy is so much smaller than the size of most biological samples. vCLEM may be used to target and track a structure between two imaging modalities, or may be used to localise molecules to structures within the volume, providing clues to their function (Fig.4).

vCLEM for tracking at the single-cell level tends to rely on external coordinate systems such as gridded coverslips for coarse targeting ^7,124^ At the tissue scale, the problem becomes more complex because of the sheer volume of the sample compared to the size of the target structure, and workflows often rely on anatomical features such as blood vessels as landmarks for correlation ^44,125–127^. One option that has been employed to great effect in large specimens is the use of high-power lasers for branding marks within the specimen or on the resin block, laying a trail of breadcrumbs to individual cells and features of interest ^23^,^44^,^109^,^128–131^.

vCLEM for molecular localization uses high-resolution correlation to overlay fluorescent microscopy images of fluorescent proteins acquired post embedding with electron microscopy images for function-structure studies. Methods have been developed that allow retention of the fluorescence of proteins after resin embedding, delivering accurate correlation of fluorescence to ultrastructure ^23,118,132–138^. The discovery that fluorescent proteins showed blinking properties within the low-vacuum environment of SEM has allowed molecular localization at super-resolution scales ^118^. A recent study has carefully optimised the use of in-resin fluorescence, resulting in a complete and robust workflow for vCLEM ^23^. A special case of vCLEM is correlative array tomography ^70–72,108,139^, where molecular information from antibody immunolabeling, fluorescent proteins or neural tracers is acquired from the same ultra-thin sections as imaged using electron microscopy, thus facilitating registration of the different modalities and enabling multiplexed molecular labelling.

## Results

### Data acquisition

All commercial electron microscopes are bundled with proprietary acquisition control software that allows users to set parameters and manually acquire images. Parameters to be optimized include voltage, probe current, field of view, pixel size, slice thickness, dwell time and exposure (depending on the imaging modality used). For vEM, additional software is needed to manage the acquisition workflow and drive the stage, the ultramicrotome or the ion beam, for example, DigitalMicrograph (3View, Ametek Inc.), Maps (VolumeScope, ThermoFisher), Atlas 5 (Zeiss) and custom software with a tablet interface (Katana; ConnectomX). To extend the capabilities of commercial software and offer more flexibility, control, specialized features and ease of use, several open-source solutions have been developed. The earliest example is SerialEM, an acquisition control software developed to perform automated electron tomography with tilt series ^140^. WaferMapper is a MATLAB software package that automates image acquisition from sections collected on wafers for array tomography ^141^. SBEMimage is an open-source Python application designed to control complex image acquisition workflows with SBF-SEM ^142^ and array tomography ^112^. piTEAM ^85^ enables large-scale TEMCA image acquisitions, with core components controlling the microscope(s), a web-based graphical user interface, and a graphics processing unit-based image processing pipeline. Automated targeting of regions of interest for FIB-SEM can be achieved with CLEMSite ^143^.

### Data processing

Data processing in vEM tends to be customized into application-specific pipelines, lifting modular components from different software platforms.

#### Volume reconstruction

After image acquisition, the series of individual images must be reconstructed into an image volume. This step may include alignment of serial images within a single volume, and subsequent stitching together of multiple image volumes, depending on the size of the original sample. Alignment of adjacent images should recover continuity across the image volume and retain the geometrical properties of the original sample, despite distortions that may occur during sample preparation and image acquisition. Cross correlation ^92,144^ and Scale Invariant Feature Transform (SIFT) ^145^ are commonly used techniques for the detection, characterization and matching of local features in image series. The SIFT concept was expanded to automatically register large image mosaics by globally minimizing the registration error, implemented as part of TrakEM2 in ImageJ/Fiji ^146–149^. Another method called Alignment to Median Smoothed Template (AMST) ^150^ was developed to align FIB-SEM datasets. Elaborating on sequential use of rigid and affine transformations, it has the advantage of correcting for local pixel size variations whilst preserving long-range alignment of structures. Following the general trend of exploiting deep learning for computer vision tasks, the largest currently available vEM volumes have been aligned through new learning-based procedures, combining defect detection, short-range and long-range alignment ^151,152^. In current practice, machine learning techniques are not used for alignment. However, there is ongoing research implementing this as a strategy ^153^.

#### Image enhancement

vEM datasets can be notoriously challenging to analyse due to their high dimensionality, texture, variations in intensity within and across volumes, noise and resolution loss due to sampling. Image enhancements to improve contrast or noise characteristics may be applied to the serial images. Traditional methods may be used to reduce noise within an image, for example contrast limited adaptive histogram equalization (CLAHE) ^154^, available as a standard image processing option in Fiji. Content-aware image restoration methods have also been applied to vEM data sets to improve the signal-to-noise ratio. In general, while supervised network training works well for SEM reconstruction, TEM data requires semi-supervised (Noise2Noise) or unsupervised (Noise2Void) training methods ^155^.

#### Multimodal registration

For vEM datasets collected as part of a correlative or multimodal workflow, an additional step is required to correlate the different datasets to each other. Here, we clarify correlation for relocation as the overlay of shared features or patterns between two or more imaging modalities, with the intention of targeting an ROI for imaging. On the other hand, correlation for registration is the accurate (and often computational) alignment of data sets from two or more modalities, with the aim of imaging cells or macromolecules that were preferentially labelled in a subset of the modalities. As an example, X-ray microscopy can reveal the position of a neuron or cell for downstream imaging by vEM, whereas overlay of fluorescence microscopy and vEM data sets acquired from the same volume can reveal the identity and ultrastructure of an organelle containing a fluorescently tagged molecule. Registration may also be used to align image volumes from different specimens from a population to create an average atlas of ultrastructure of a model organism ^156,157^.

To register multimodal data, a fiducial of some sort is required. Fiducials are often specific to - and constrained by - the sample type and biological question. With array tomography datasets, where different modalities come from the same ultra-thin sections, registration of fluorescence microscopy and vEM can be done with high precision in 2D, and thus in 3D, and cell nuclei are often used as fiducials ^108^. In vCLEM studies, mitochondria ^35,158^ and nuclei ^50^ are popular internal fiducials, as they are easily labelled with chemical dyes or fluorescent proteins and can generally be recognized in EM images by their morphology. Software packages such as 3D slicer, eC-CLEM ^159^, BigWarp ^160^ and elastix ^161,162^ offer varying degrees automation for multimodal image alignment.

### Image segmentation

#### vEM image segmentation

Image segmentation - the detection and delineation of structures of interest - is required for extraction of quantitative information from rich vEM datasets. Non-discriminant contrast, diversity of appearance of structures, and large image volumes turn vEM segmentation into a highly non-trivial problem, where cutting-edge methods relying on state-of-the-art computer vision techniques are still far from reaching human parity in segmentation accuracy. Although significant progress has been made in recent years, largely due to the introduction of deep learning-based methods, there is not yet a single reliable and easy-to-use solution for fully automated segmentation of vEM images. Practitioners must choose between (or combine ^163^) manual, semi-automated and fully automated solutions based on the difficulty of the segmentation problem, the data size and the computational expertise and resources of their team or institution. Furthermore, almost all automated solutions rely on machine learning and may require large amounts of example segmentations to train a model, although in some cases models trained for the same task on similar datasets are available and can be applied directly.

#### Manual and semi-automated segmentation

For small datasets, for subsets of large datasets, and for datasets in which only a few structures are to be analysed, manual or semi-automated approaches are often preferable. Here, ready-made solutions that do not require computational expertise or example segmentations are available. Open-source solutions exist: the ilastik carving workflow ^164^ enables users to sequentially extract objects from a volume by labelling parts of the image that belong to the object and the background (everything else) and using a region growing algorithm for segmentation. Microscopy Image Browser (MIB) ^165^ also contains a set of tools for semi-automatic segmentation based on thresholding, morphological image operations and region-growing. The software suite IMOD ^166^ provides manual and semi-automatic drawing tools for tracing and refinement of organelles. Amira (ThermoFisher Scientific) and Imaris (Oxford Instruments) are commercial software packages that offer semi-automated segmentation combined with advanced visualization capabilities. The Fiji plugin TrakEM2 ^147^, Reconstruct ^167^, Webknossos ^168^ and VAST ^169^ are also used. Note that these tools can often only load data up to a certain size. To overcome this limitation, one can downsample the data if the structures of interest are discernible at lower magnification, or crop regions of interest. Downsampling or cropping can be achieved with Fiji, for example using the BigDataProcesser plugin ^170^, or programmatically for even larger data using Dask or other libraries that provide easy access to task parallelization. These tools only support a certain set of image data formats. Bioformats ^171^, which is integrated within Fiji, can be used to convert between all common data formats.

#### Automated segmentation

If the data size exceeds the capabilities of semi-automatic segmentation, for example when structures of interest need to be fully and densely segmented in a large volume, fully automated methods must be employed. These methods are almost universally based on machine learning: they use example data in the form of ground-truth segmentations to estimate parameter values for the segmentation model, which can range from a simple classifier (rarely used in vEM due to the complexity of the task) to a very deep neural network with millions of parameters. Initially, fully automated segmentation was developed for connectomics. This application requires the complete segmentation of neurons and synapses in large volumes and is thus only feasible with a high degree of automation. Development has been significantly advanced through open challenges (such as ISBI2012 ^172^) leading to methods that enable circuit reconstruction for the brains of small organisms ^173^ or brain regions ^91^. Recently, large-scale segmentations have also been achieved in other domains such as cells and selected organelles in a complete small organism ^50^ or many organelles in cells ^99,158^.

Machine learning methods can be trained to produce different flavours of segmentation, labelling the pixels either by semantics (for example, label all mitochondria pixels as 1 and the rest as 0) or by the objects they belong to (for example, label all pixels of the first mitochondrion as 1, of the second mitochondrion as 2, of the *n*th mitochondrion as *n*, with non-mitochondrion pixels as 0). Furthermore, a semantic segmentation can be transformed into an object segmentation by connected component labelling or a watershed transform. This approach is often used for separated objects such as mitochondria or nuclei and can be found, for example, in the Fiji plugin MorphoLibJ ^174^. Alternatively, semantic segmentation can be used to predict boundaries between objects. The object segmentation is then achieved by a watershed transform or graph-based agglomeration. This approach, often used to segment cells or densely packed vesicles, is also available in MorphoLibJ or in the ilastik boundary-based segmentation workflow ^164^. Finally, methods that directly predict an object segmentation have now been introduced for light microscopy ^175,176^ and, very recently and only in the form of research code, for vEM data ^157^.

**Figure 5:**
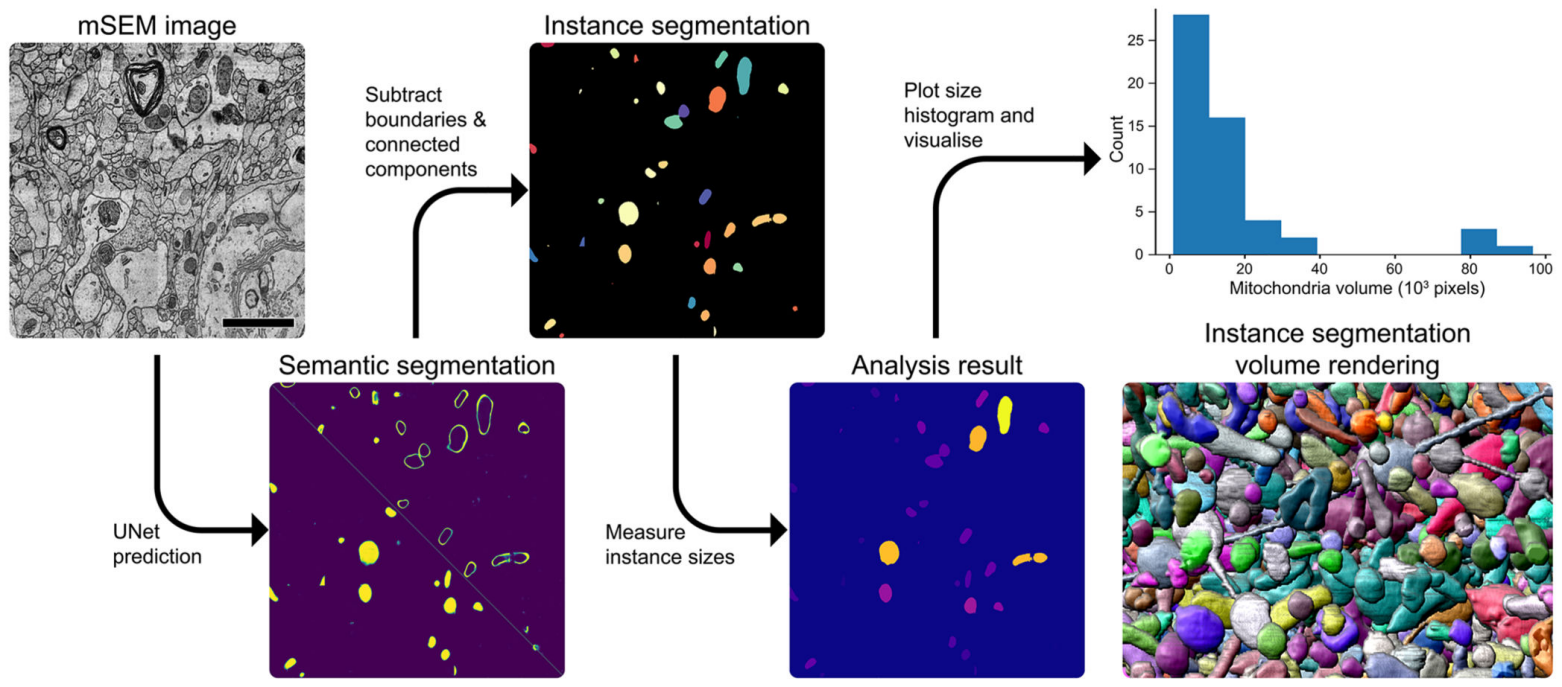
A typical vEM image analysis pipeline. In this example, mitochondria have been segmented from an array tomography image stack acquired using multibeam SEM (mSEM) (MitoEM data challenge). The analysis pipeline contained the following steps: mitochondria pixel probabilities and boundaries were predicted with a pretrained UNet, boundary probabilities were subtracted from mitochondria probabilities and connected components applied to achieve an instance segmentation. Size per instance was measured and projected to the image and, finally, the extracted mitochondria volumes were plotted as a histogram. All analysis was performed in three dimensions. An example volume rendering of the instance segmentation is also shown, demonstrating number and complexity of mitochondria in a sub-volume of the data set. Scale bar in mSEM image = 2 μm. mSEM image courtesy of the Lichtman lab at Harvard University.

Generally, machine learning-based segmentation models can be divided into two categories: feature-based learning and deep learning. Feature-based learning methods use a set of predefined features (usually linear and non-linear image filters) as input to a non-linear classifier such as a support vector machine or a random forest that outputs the (semantic) segmentation. They need few examples and are available via user-friendly tools. Methods using deep learning do not rely on pre-computed features but, instead, learn features and segmentation jointly. They can solve more difficult segmentation problems, but their superior accuracy requires much larger amounts of examples, and the training must be performed on graphics processing units. The U-Net ^177^ is a widely used deep learning model architecture for segmentation in vEM, employed as a backbone in most state-of-the-art segmentation methods (Fig.5).

Feature-based segmentation methods can be trained interactively from brush-stroke annotations and are implemented in user-friendly tools such as the ilastik pixel classification and autocontext workflows ^164^ and the Fiji Weka plugin ^178^. Training deep learning models is more challenging than feature-based models, as these require significantly more training data, computational expertise and resources. Consequently, most user-friendly tools delegate the training step to method developers and implement prediction with pretrained models for end users. These tools include DeepImageJ ^179^, the ilastik neural network workflow ^164^, DeepMIB ^180^, CDeep3M ^181^, epanada ^182^ and APEER (Zeiss). For successful application, the model needs to be trained on data very similar to the data at hand, preferably using generalizability-enhancing tricks such as data augmentation, while unsupervised pretraining on large unlabelled and heterogeneous datasets may increase model performance overall ^183^. All tools listed above provide a set of pretrained models and the recent Bioimage Model Zoo community project hosts models in a cross-compatible format that is currently supported by DeepImageJ, ilastik and ZeroCostDL4Mic ^184^. While some of these tools (CDeep3M, APEER) also implement network training, they do not eliminate the need for many segmentation examples and a graphics processing unit. Given these requirements, for practitioners with some computational expertise it may be easier to train a new model directly using Python scripts, which provides more flexibility. An intermediate solution is the use of prepared Jupyter notebooks such as those provided by ZeroCostDL4Mic ^184^ that come with a running environment and use publicly available computational resources.

Efficient training and post-processing procedures for deep learning methods constitute an active area of research. Most of these methods are made available as Python code using one of the most popular deep learning frameworks (PyTorch or TensorFlow), and they can be retrained, extended and applied through programming in Python. Mala ^185^, MWS ^186^ and SegEM ^187^ were originally developed to segment neurons but can be used for other segmentation tasks as long as objects have discernible boundaries. The approach of flood filling networks ^188^ has been used for many large-scale connectomics segmentations ^116,173^, and synapse detection and synapse partner prediction tasks ^189,190^. Training deep learning methods for new data requires many segmentation examples; large and heterogenous training datasets may produce more generalist models ^182^. These examples can be generated using the manual/semi-automated tools introduced earlier or more general-purpose annotation tools such as napari ^191^ and Fiji plugins (like LabKit). Alternatively, proofreading tools can be used to correct an initial segmentation, for example FlyWire ^192^, Paintera ^193^ or Dojo ^194^. These tools enable faster generation of examples, but generally have a steeper learning curve and less documentation. They can also be used to correct errors in the final segmentation results.

Although the methods listed above can be wrapped in scripts and notebooks to be made accessible to practitioners of limited computational experience, application of these methods to large data sets, from tens of gigabytes to hundreds of terabytes, requires significant computational resources and experience with large-scale distributed computing. For such teams, open-source solutions for large-scale deployment exist and can be freely exploited ^188,195–197^.

#### Commercial solutions and crowdsourcing

If the segmentation problem at hand is not feasible with the tools introduced above, for example owing to missing pre-trained models or lack of computational expertise, the task can also be out-sourced to companies offering segmentation as a service, for example scalable minds or ariadne.ai. For large projects, crowdsourcing of the segmentation effort is also an option, which has successfully been pioneered for segmenting retinal neurons ^198^ as well as cells and organelles ^199^, and is currently underway for neurons in the fruit fly brain ^202^.

#### Examples of image segmentation workflows

Associated with this Primer, we provide two example data analysis workflows for neuron and mitochondria segmentation. The first example demonstrates how to use a pretrained network for neuron boundary segmentation in ilastik, followed by graph-based post-processing to obtain an instance segmentation. The second example demonstrates mitochondria segmentation for a large electron microscopy volume using a pretrained network and Python libraries. For both examples, alternative approaches are sketched.

### Data exploration and visualization

Interactive visualization of large vEM datasets is challenging. Important features for visualization software are the ability to view the data from arbitrary (non-axis-aligned) angles, surface mesh rendering for displaying segmentation results, and support for viewing large datasets. Many of the software platforms already mentioned provide options for data visualization and exploration, including Fiji, napari, IMOD and MIB. However, exploration of very large datasets is not possible with many visualization tools, because they rely on loading all data into memory or only provide inefficient access to data out of memory. To overcome these limitations, support for chunk-based data access and a precomputed multi-scale image pyramid format is necessary and is offered by some microscopy vendors. In addition, support for on-demand access of data stored in the cloud is desirable. In Fiji, the BigDataViewer ^200^ and MoBIE ^201^, plugins can be used for exploring large data. For collaborative skeleton tracing and connectome analysis, Catmaid ^202^, Neuroglancer ^203^ and tools from the Knossos family (Knossos ^204^, pyKnossos ^125^ and webKnossos ^168^) provide similar functionality and extend support to collaborative annotation for volumetric segmentation.

## Applications

Conventional 2D imaging with TEM requires the trained eye of an expert to interpret the intricate topology of organelles within the crowded environment of a cell. A particularly elegant, and accurate, interpretation of the sarcoplasmic reticulum was made by Porter and Palade in 1957 ^205^. In this section, we have selected a few examples to illustrate the breadth of vEM applications following a scale logic, from single organelles through to cell-cell interactions, small organisms, and tissues. We do not include applications in the physical sciences, but it should be noted that FIB-SEM techniques, in particular, are commonly used in the semiconductor and materials sciences. This list of applications is far from being exhaustive, and we encourage readers to perform literature searches to find examples of vEM data representing their own organelles, cells and tissues in this fast-evolving field.

### vEM within a single cell

#### The cytoskeleton

**Figure 6:**
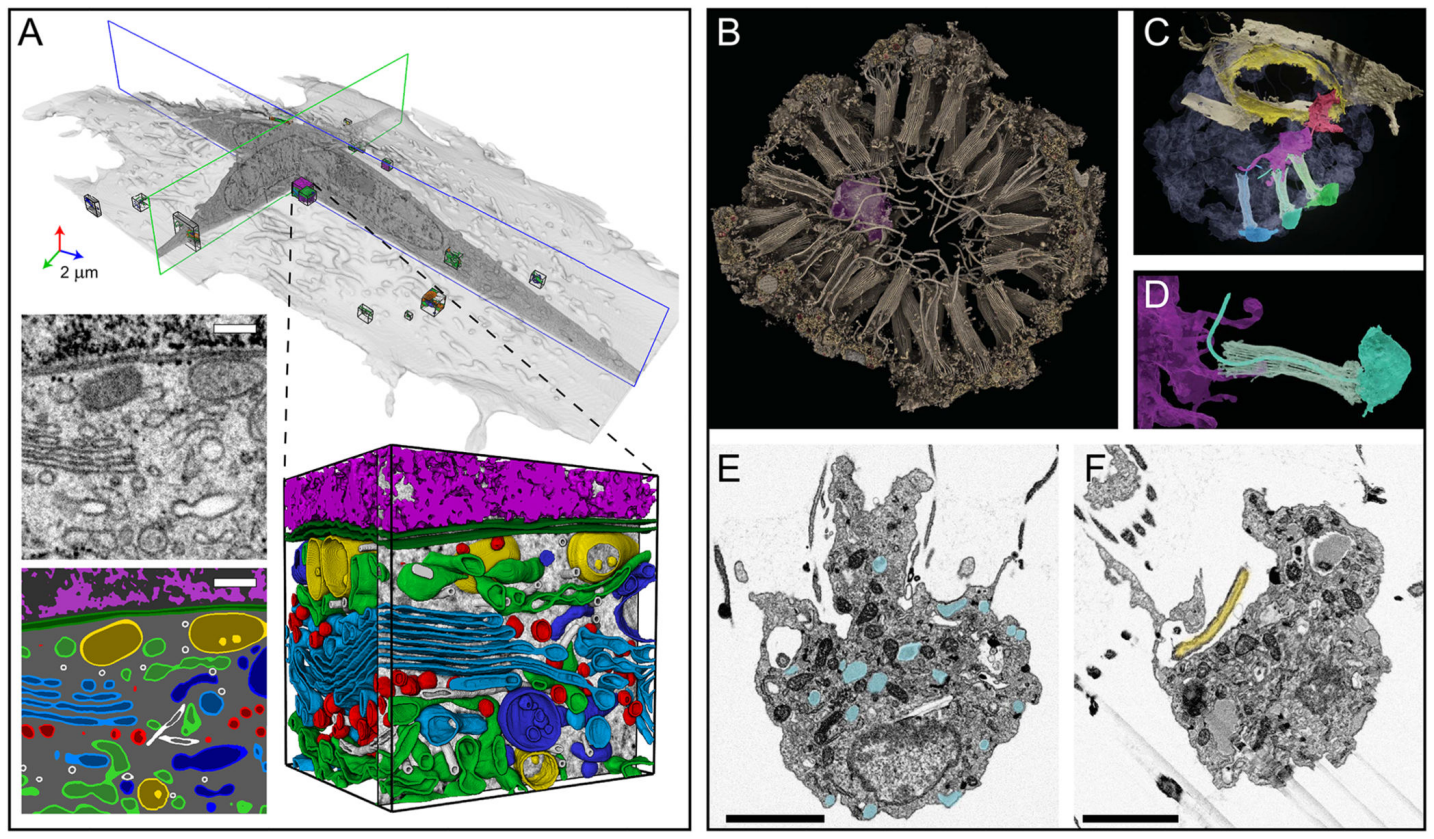
Examples of multiscale vEM on organelles and cells. **A:**Segmentation and analysis of organelle volumes in a HeLa cell data set, acquired using enhanced focused ion beam scanning electron microscopy (eFIB-SEM) ^150,158^. Fifteen manually annotated training blocks were produced from a whole cell to train deep learning models to classify different organelles within the volume. Shown are a 3D rendering of one training block (right inset, 1.2 × 1.2 × 0.95 μm), with a single eFIB-SEM slice (top left inset), and annotation of every voxel within this slice (bottom left inset). Scale bars for insets = 200 nm. **B-F:** Analysis of cell diversity and nervous system evolution in sponges, data sets acquired using FIB-SEM to highlight neuroid-choanocyte interactions ^46^. 3D volume of entire choanocyte chamber segmented using machine learning with a neuroid cell, previously identified using light microscopy (violet), and interacting with multiple microvilli within the chamber (**B**); segmented volume showing the violet neuroid cell, and a second neuroid cell (red) closer to apopylar collar, contacting cilia and microvillar collars of three choanocytes (blue, turquoise and green) and apopylar cells (yellow) (**C**); higher magnification view of segmented neuroid cell (violet) with filopodia extending into microvillar collar (turquoise) (**D**); single slice from FIB-SEM data set showing a neuroid cell with secretory vesicles highlighted in cyan. **F:** Single slice from the same FIB-SEM dataset showing neuroid cell forming a pocket around tip of a choanocyte cilia (yellow) (**F**). Considered with other structural features, these findings are suggestive of a role for neuroid cells in bacteria and debris clearance and intercellular communication. Scale bars (E, F) = 2 μm. vEM, volume electron microscopy. Panel A adapted from Heinrich et al. ^158^. Panels B-F reproduced from Musser et al. ^46^.

Most cytoskeletal elements and organelles span greater distances than the typical thickness of a TEM section (30 to 100 nm), and therefore require vEM to be seen in their entirety (Fig.6). Yet, they are usually challenging to detect by electron microscopy as their cross-sectional dimensions are often close to the minimum pixel size of the given acquisition technique. Microtubule spindles, for example, have been reconstructed using ssTEM tomography ^206^ at sufficient resolution to count microtubules, measure their length and visualize how they interact with the kinetochore (resolution of 2.3 nm). However, the acquisition and processing of serial tomograms, and, most importantly, the segmentation of individual microtubules, represent a major effort that very few laboratories have undertaken to date. More recently, state of the art FIB-SEM data at 4 nm isotropic resolution have reaffirmed the extent of single microtubules in cultured cells ^158^ and pancreatic β-cells ^163^, illustrating how microtubules connect with multiple organelles.

#### Secretory organelles

Organelles of the secretory pathway have been studied in 3D with many vEM techniques. The complex architecture and connectivity of stacked endoplasmic reticulum sheets in rodent neuronal and secretory cells was unravelled by Terasaki et al. ^207^ using array tomography. Importantly, the section thickness (30-40 nm) was set to be lower than the ER lumen diameter and the inter-stack spacing. The changing morphology of the endoplasmic reticulum during cell division was studied by ssET and SBF-SEM, with or without electron-dense DAB labelling of the ER lumen ^208,209^. Further endoplasmic reticulum studies drove the introduction of image analysis tools to capture and model the complexity of the organelle ^165,210^, including a description of the curvature landscape that defines the various endoplasmic reticulum sub-compartments in cultured cells ^158^. The endoplasmic reticulum morphology of specialized cells such as neurons has also been investigated by SBF-SEM and FIB-SEM ^211^, and studies of endoplasmic reticulum exit sites using FIB-SEM unravelled the tubular (rather than vesicular) nature of this intermediate compartment in insect ^212^ and mammalian cells ^213^.

Further down the secretory pathway is the Golgi, the complexity of which justifies the need for 3D visualization. In the early 2000s, a detailed description of the Golgi complex visualised by ssET was published ^77^. ssET is still the method of choice to study this organelle as the distance between cisternae in a stack requires high-resolution imaging. More recent studies capitalized on the larger acquisition volumes offered by FIB-SEM to capture the full stretch of the Golgi ribbon ^99,163^. Further, the endo/lysosomal pathway has been extensively imaged by vEM, using vCLEM to assign molecular identity to otherwise ambiguously shaped structures ^214,215^.

#### Other subcellular components

vEM has made significant contributions to the study of inter-organelle interactions ^216^, identified by spatial proximity (< 20 to 30 nm) between the limiting membranes of two organelles. In cultured cells ^158,163^ and liver tissue, FIB-SEM has shown changes in organelle morphologies associated with changing physiological states ^217^. vEM is also the technique of choice to describe the complexity of pathogen-induced structures, such as virus replication organelles ^8,218–220^ and membrane perturbations associated with viral budding in HIV and SARS-CoV-2 infected cells ^100,221–223^.

### vEM of cell-cell interactions

#### Cell-cell interaction in metazoans

At the cellular level of our scale logic, vEM workflows are ideally suited to studying the finer details of the complex interactions between individual cells and/or groups of cells. For instance, in *Spongilla lacustris*, a freshwater sponge, vCLEM was used with FIB-SEM to demonstrate that individual neuroid cells interact with multiple choanocytes, supporting a role in neuron-effector communication, and with their conserved genetic makeup, as putative neuronal ancestors ^46^. Using array tomography and SBF-SEM, fibre cells in the Placozoa *Trichoplax adhaerens* were shown to contact multiple adjacent cell types and display evidence of phagocytic activity, suggesting a macrophage-like role in innate immunity that appeared early in metazoan evolution^224^. In symbiotic microalgae (the algal symbiont *Phaeocystis cordata*, in its acantharean host), reconstructions of FIB-SEM image series showed significant alterations in morphometric measures, reflecting interaction between the symbiont and controlling host ^225^. Similar approaches have also been used to examine conserved morphometric features in various phytoplankton, suggesting that topology could be modulated by energy-management constraints ^226^.

#### Host-pathogen interactions

vEM techniques are also very powerful for studying host-pathogen interactions ^227^. vCLEM studies combining fluorescence microscopy and FIB-SEM have shown how *Mycobacterium tuberculosis*, first confined within a membrane-bound phagosome, could escape to the cytoplasm of their host macrophage ^228,229^. A multimodal vCLEM approach was used with ssTEM, SBF-SEM and FIB-SEM to validate zebrafish as a model system for studying *Toxoplasma gondii* infection, showing that macrophages have a role in parasite clearance in vivo ^12^. Likewise, FIB-SEM revealed the complex structure of *T. gondii* rosette formation and connectivity ^230^, and cell-cell interactions at the virological synapse in HIV infection ^221^.

#### Immune cell-cancer interactions

The whole cell volume of a mouse cytotoxic T lymphocyte forming an immunological synapse with an ovarian cancer cell was revealed using FIB-SEM ^99^; the topology of this system was previously difficult to study in detail. In more complex multicellular organisms, intercellular communication is crucial, and one such mediator of crosstalk and material transfer is thought to be tunnelling nanotubes. Using vEM, tunnelling nanotubes have been shown to play a role in the stroma-mediated protection of leukemic cells through cell-cell vesicle trafficking ^231^, and microtubule bridges connecting cells in the early mammalian embryo have been shown to facilitate membrane transport between cells ^232^.

#### Nerve cell interactions

vEM has been widely used to examine cell-cell interactions in neuroscience. In the mouse brain, ssTEM showed that ultrastructural features of the blood brain barrier, including increased pericyte-brain endothelial cell contact, are altered during aging ^233^, and single axons form clustered compound multi-synaptic contacts of distinct morphologies with individual neurons, thus maximizing their influence on the postsynaptic neuron ^139^. FIB-SEM has been used to demonstrate that some primary cilia form serotonergic axo-ciliary synaptic contacts in the hippocampus ^234^. SBF-SEM was used to reconstruct two individual cells from a large dataset of a leech ganglion, discovering new connections ^235^. Inferences were also made about previously unstudied pathways, validated by post hoc comparative electrophysiological measurements in matched living tissues. Tran et al. ^236^ demonstrated an elegant co-culture system, designed to examine neuron-neuron interactions in detail with FIB-SEM and ssTEM. Using array tomography, synaptic inputs and outputs of an inhibitory interneuron were mapped ^237^.

### vEM at the organism and tissue scale

**Figure 7:**
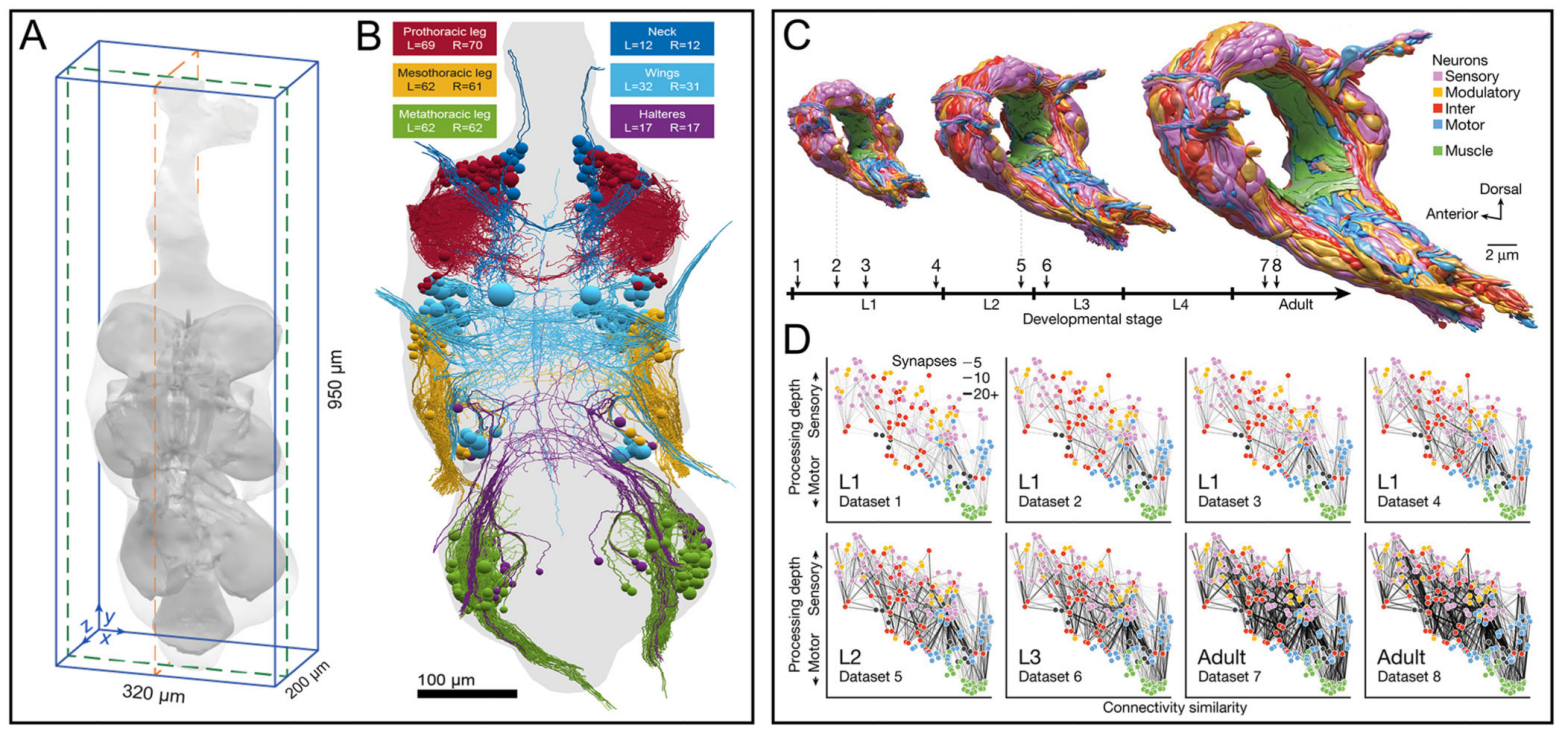
Examples of multiscale vEM on model organisms and connectomes. **A:** Volumetric rendering of a *Drosophila melanogaster* ventral nerve cord (VNC) data set ^84^ showing the full extent of all imaged tissue (light grey) and the outline of the VNC neuropil (dark grey), with overall dimensions indicated. Data set acquired using GridTape transmission electron microscopy (TEM). **B:** All motor neurons in the thoracic segments of the VNC were reconstructed and analysed. Each motor neuron projects an axon to one peripheral nerve, leaving the bounds of the electron microscopy dataset, to innervate muscles. Cell bodies are represented as spheres, coloured along with their projections, in accordance with the target of innervation. **C:** Volumetric models of *Caenorhabditis elegans* brain ^238,250^, coloured by cell type, and shown at three stages in developmental timeline. The datasets were acquired using both serial section TEM (ssTEM), and single-beam array tomography with automated tape-collecting ultramicrotome (ATUM), and manually reconstructed using CATMAID. **D:** Wiring diagrams highlighting changes in complexity of connectivity during brain development for eight individuals indicated on the timeline in C. Each circle represents a cell, again coloured in accordance with cell type and target of innervation, and each line represents a connection between two cells with at least one chemical synapse. Vertical axis denotes signalling from sensory perception (top) to motor actuation (bottom). Horizontal axis denotes connectivity similarity; neurons that share partners are positioned more closely. Signal flow and connectivity similarity are based on accumulated connections from all datasets. Although brain geometry does not change substantially during development, with neuron number and position being fairly constant, complexity and relative strengths of synaptic connections evolve significantly during development. vEM, volume electron microscopy. Panels A and B adapted from Phelps et al. ^84^. Panels C and D reproduced from Witvliet et al. ^250^.

**Figure 8:**
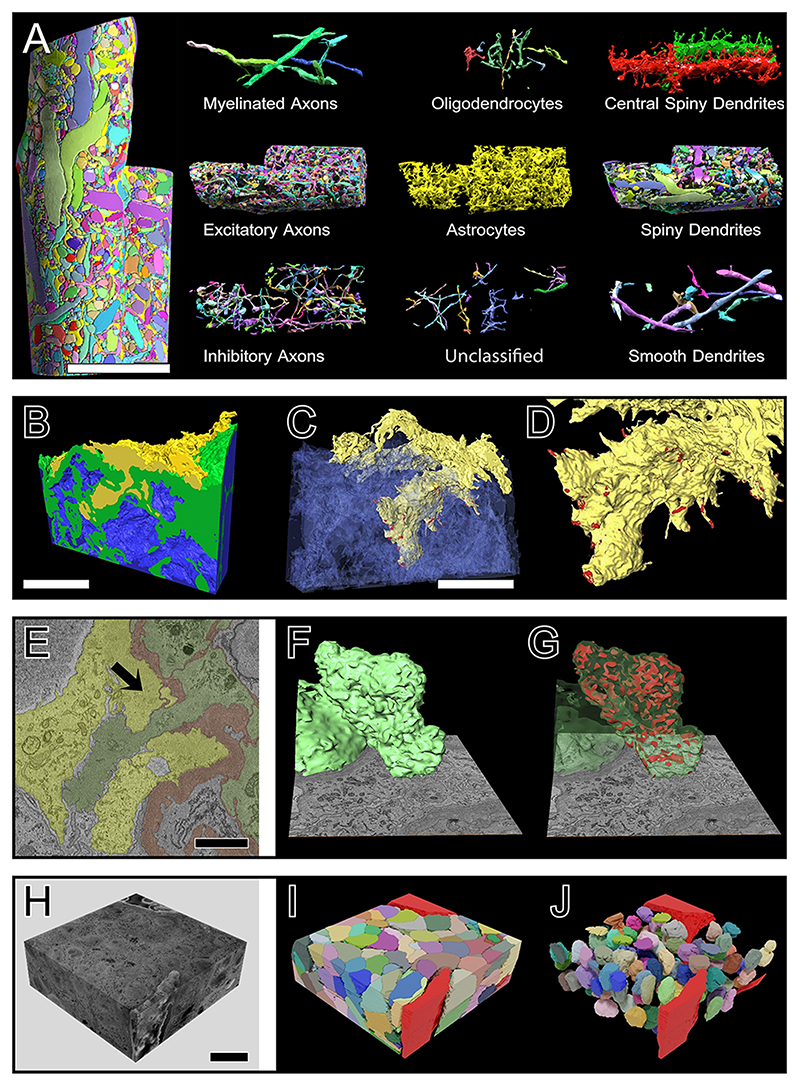
Examples of multiscale vEM in tissues and the clinical setting. **A:** Analysis of a 1,500 μm^3^ volume of mouse neocortex, generated using array tomography with automated tape-collecting ultramicrotome (ATUM), imaged using single-beam scanning electron microscopy (SEM), and segmented automatically ^254^. The segmented volume was mined to study cellular composition and connectivity. The saturated reconstruction volume (left) had a varied composition which could be divided into individual subcategories, including cell types, axons, dendrites, and other unclassified processes. Scale bar = 7 μm. **B-D:** 3D ultrastructure of a kidney biopsy specimen from a patient with lupus nephritis studied using serial blockface SEM (SBF-SEM) ^261^. In this 3,700 μm^3^ data set, the glomerular basement membrane was found to be disrupted, with podocyte cytoplasmic processes (yellow) extending into the glomerular basement membrane and mesangial matrix (green) (**B**). Reconstructed in three dimensions, the podocyte cytoplasmic process (yellow) displayed highly complex topology with multiple spikes extending towards surrounding mesangial cells (blue), forming more than 100 contact sites (red) (**C,D**). Scale bars = 10 μm. **E-G:** SBF-SEM analysis of human biopsy samples from patients with immunoglobulin A nephropathy ^262^ highlighted the disruption of the glomerular basement membrane (red) and penetration of mesangial cellular processes (green) into the urinary space (highlighted by a black arrow with podocyte highlighted in yellow) (**E**); 3D reconstruction of the penetrating mesangial cell (**F**) revealed multiple contact sites with podocytes within the urinary space (red) (**G**). Scale bar = 1 μm. **H-J:** To study the 3D organization of patient- derived hepatoblastoma xenografts, SBF-SEM was used 267: one SBF-SEM data set (**H**) was used to develop a semi-automatic segmentation procedure, revealing 182 tumour cells (**I**) and 113 nuclei (**J**) within the volume, from which bioarchitectural parameters could be extracted to further study tumour tissue architecture. Scale bar = 20 μm. vEM, volume electron microscopy. Panel A reproduced from Kasthuri et al. ^254^. Panels B-D reproduced from Takaki et al. ^261^. Panels E-G reproduced from Nagai et al. ^262^. Panels H-J reproduced from de Senneville et al. ^267^.

vEM reveals principles of tissue organization that are impossible to capture with 2D approaches or with lower resolution methods. For example, array tomography of mouse ovarian follicles uncovered previously unknown complex interactions between different cell types via cytoplasmic extensions and gap junctions ^238^. vEM has now been adopted for tissue scale investigations across species (Fig.7), including in plants ^239,240^, and a variety of insects ^241,242^ and other arthropods ^243^. vEM of the nervous system, in particular, has rapidly advanced in recent years, because mapping neuronal circuits at synaptic resolution is considered essential to understanding how the brain works and how specific behaviours are generated ^244^ (Fig.8). Thus, within the past 10 years an impressive number of large vEM datasets of brain tissue have been produced, including the full or partial connectomes of the mammalian retina ^73,245^, ascidian tadpole larva *Ciona intestinalis* ^246^, the larva and adult Drosophila brain ^82,173,247^, zebrafish brain ^13,31^, a cubic millimetre of mouse brain ^248^, a cubic millimetre of human brain ^116^ and the pathoconnectome of a degenerating human retina to study neurological diseases ^249^. This list continues to grow at pace, and it is now possible to image the connectomes of several individuals from the same species (*C. elegans)* to address questions of development and plasticity ^250^. Peripheral nervous system investigations have also benefited from vEM, for example by understanding the structure and connectivity of cell types within the mouse circumvallate taste bud ^251^. For smaller organisms, vEM of the entire body *(Platynereis* larvae ^252,253^) enables investigations of the structure and function of the nervous system in the context of the whole body and its interactions with the environment.

In addition to addressing the research question that initially motivated their collection, such large vEM datasets have also proven to be a valuable resource for addressing unrelated questions at various scales. For example, a vEM dataset from mouse cortex was generated^254^ to investigate the rules of neocortical synaptic connectivity, and has also been used to define the 3D organization of the endoplasmic reticulum ^255^, the structure of mitochondria ^256^ and the distribution of myelin in axons of neocortical pyramidal neurons ^257^.

### vEM in the clinical setting

Electron microscopy has long been used as a tool in the clinic, most commonly in diagnosis of ciliopathies and in renal pathology ^258^. Diagnostic electron microscopy generally uses TEM to deliver the resolution needed to measure defects in, for example, the central complex and microtubular arrangements in cilia in primary ciliary dyskinesia ^259^, and glomerular structures including the basement membrane in the kidney. A recent move has seen several teams investigate the potential of vEM techniques to reveal additional diagnostic features in volumes greater than that of a single ultra-thin section. Doubling the imaged volume using dual-axis electron tomography and sub-tomogram averaging demonstrated that a reduction in proximal outer dynein arm volume could be detected in archived specimens from patients with primary ciliary dyskinesia with a mutation in the *DNAH11* gene, that could not be seen using standard ultrathin sections ^260^. Other work has demonstrated that SBF-SEM can reveal disruption of the glomerular basement membrane and associated changes in podocyte cell contacts in human biopsy samples from lupus nephritis ^261^ and patients with IgA nephropathy ^264^. In these cases, image volumes and segmentation were essential to trace and capture penetrations of cells through the basement membrane and rare cell-cell contact events. vEM techniques are now finding broader application in clinical research, to study synapse organization in post-mortem brain from patients with Alzheimer’s disease ^263^ and organelle changes in Parkinson’s disease ^264^, the tumour microenvironment in cancer biopsy samples ^265–267^, cell-cell contacts in the alveolar epithelium of the lung using archival material ^268^ and the osteocyte and lacuno-canalicular network in osteoarthritic and osteoporotic human bone ^269^.

## Reproducibility and data deposition

Biological electron microscopy is still largely a manual discipline. Samples are typically prepared using customized protocols tailored to the research question and qualitative observations are often made on a small number of hand-selected images. This approach has led to seminal discoveries in biology yet limits throughput and complicates reproducibility. However, recent community efforts aim to produce data and tools that will support reproducibility in vEM.

### Factors influencing reproducibility

The vast heterogeneity of biological samples and applications preclude a universal vEM sample preparation protocol, as evidenced by the daunting number of published protocols. Reproduction is confounded by historical differences in protocols between laboratories; the number of steps in sample preparation workflows, which deters users from optimizing adequately working protocols; variation in reagent batches; a lack of standard parameters and quality control metrics; and the absence of a repository of reference protocols with corresponding images showing examples of data of varying quality. To address these issues, reference protocols and consensus guidelines on optimization of workflows must be agreed within the expert vEM community. The production of benchmark specimens from different laboratories using these reference protocols will allow assessment of local variables. Public availability of these data will allow crowdsourcing or artificial intelligence approaches to derive quality control scores. Increased throughput of sample preparation using microwave processing and robotic devices will also support reproducibility.

The community currently lacks a standard set of quality control checks for the microscopes used to acquire vEM data, but inspiration can be taken from the introduction of guidelines and standards for quality control by the QUAREP-LiMi consortium in light microscopy ^270^. Empirical and heuristic choices made during image acquisition, plus (hidden) differences in the state of the instrument mean that imaging runs are rarely reproducible between laboratories. Benchmark specimens prepared from reference protocols would allow acquisition of benchmark datasets, which could be publicly deposited and used to better understand differences between microscopes and imaging parameters. Fixing parameters could result in reliably repeatable acquisition runs. Environmental stability during long imaging runs is critical for quality and reproducibility, and accurate monitoring of section thickness would improve the fidelity of downstream image volume reconstruction. Automation of imaging and internal quality control steps, such as on-the-fly focus and stigmation corrections, and feedback loops to maintain spatial fidelity are critical aspects of vEM ^52^. Lack of full reporting of image acquisition parameters has historically limited the reproducibility of vEM experiments, and therefore community consensus on parameters to be reported for each vEM imaging modality would provide valuable guidance and extend the recently published REMBI minimal metadata recommendations to support reproducible vEM data acquisition ^271^.

A lack of metrics for vEM data means that quality control and optimization are based on manual visual inspection. To improve this, the vEM community can take inspiration from the work of the cryo-EM community on gold standards ^272^. As with sample preparation, there is no silver bullet software package that can seamlessly process and analyse all vEM datasets. Most researchers use a mix and match/trial and error approach to visualize and mine their image data, slowing optimization and complicating reproducibility. Community guidelines on reporting methods for processing and analysis of vEM data are critical for reproducibility of published work ^271,273^. Image-to-image and multimodal registrations should be published and/or publicly deposited with accompanying landmark and transformation files for reproducing such registrations. Segmentation is still largely a manual process, despite progress in the development of machine learning algorithms, and known inter-expert variations on segmentation tasks raise questions around the fidelity of ground truth ^274,275^. Inter-expert variation is often due to inadequacies in the datasets; for example, sections may be too thick to resolve very thin axons, or to identify synapses that are sectioned parallel to the postsynaptic density, and missing sections and specimen contamination contributes to errors. Further development of machine learning approaches will succeed only with parallel improvement of data quality. Publication of usable models with accompanying weights within containerized workflows will allow reproduction of inferences.

### Data handling and deposition

Community surveys suggest that many labs are collecting vEM datasets in the 10-100 GB range, with images saved to disk in proprietary or tiff file formats, transferred to a server and downloaded by users for downstream analysis using desktop software programs as cropped or binned image stacks. However, as vEM datasets reach the terabyte regime, this approach breaks down and data handling must be carefully planned with the support of scientific computing specialists. Institutions hosting vEM technology must consider related computational expenses and hire dedicated personnel to optimize and streamline data handling. Traditional file formats, such as mrc and tiff, fail at the terabyte scale and next generation file formats ^276^ are being developed that dynamically access chunks of data via cloud storage and compute, bypassing the need to download entire datasets. Public deposition of image data and associated metadata in image archives such as EMPIAR ^277^ and the BioImage Archive ^278^ are critical in driving full analysis and reanalysis of rich vEM datasets by a distributed user base, as well as aiding development of automated image analysis tools and supporting meta-studies into reproducibility.

## Limitations and optimizations

As may be expected in a nascent field, there are currently a range of limitations affecting vEM, giving rise to an exciting and dynamic technology research, development and innovation environment. Stable, supported and accessible solutions will help overcome these obstacles and drive the adoption of vEM by the wider biological community.

### Sample preparation

Ideally, cells and tissues would be cryofixed (to ensure that dynamic events are captured) and imaged at cold temperature (to avoid shrinkage and warping artefacts due to dehydration). However, the limitation in the depth of vitrification of biological samples is ~200 μm, far below the size of most samples destined for vEM. Serial imaging of small volumes (<10 μm) of vitrified samples is possible using cryo FIB-SEM ^279,280^ or cryo soft X-ray tomography ^283^. Reassuringly, images of 3D ultrastructure from cryo soft X-ray tomography show that most vitrified cell structures have a similar appearance to those seen in room-temperature vEM, suggesting that shrinkage artefacts will not invalidate interpretation for many biological questions. A further complication of working with human tissue samples for vEM is the incompatibility of clinical processing (wax embedding and snap freezing) with good ultrastructure, and the deterioration of ultrastructure of post-mortem samples prior to fixation, a complication that was critically evident in the early stages of the COVID-19 pandemic ^282^. A lack of protocols for introducing immunolabels deep into tissue without membrane-destabilizing permeabilization techniques (methanol, Triton and so on) ^69^ also hinders molecular localization in tissues, while high-contrast genetic probes that are still visible with multiple layers of heavy metal staining are only now being developed.

### Imaging

vEM, similar to most imaging modalities, falls foul of the trade-off between speed of image acquisition, resolution of the images and the volume that can be imaged. Brute force speed-up of image acquisition results in lower signal and image quality, whereas targeted imaging may obscure important tissue context, and automated imaging of ROIs may lower the chance of serendipitous discovery by biasing acquisition. Long imaging runs and parallelization by installation of multiple instruments are possible but expensive and, currently, only implemented in a handful of dedicated facilities worldwide ^52,85^.

### Data analysis

vEM data analysis is still a massive bottleneck, being largely manual, and therefore time and resource intensive. The volume that can be analysed and the number of features that can be extracted are limited. Furthermore, most vEM analysis workflows require some level of bespoke customization, depending on the research question, sample type, sample preparation, imaging modality and quality and format of the acquired data. A mishmash of constantly evolving open-source, in-house and commercial software packages add complexity for non-expert end users. The application of machine learning will alleviate some of these analysis burdens, but universal models are not yet a reality. The infancy of the field demands significant computational literacy in researchers to create or tweak algorithms and track and correct errors. vEM data analysis is a research area in flux, but a strong push toward open-source solutions will ideally prevent siloed computational solutions.

## Outlook

The development of new tools and technologies has underpinned great advances in our understanding of how cells, tissues and organisms function in health and disease. Electron microscopy has been central to these advances, being the only technique that can reveal the structure of cells and tissues with the contrast and resolution to distinguish membranes and membrane bilayers. Whereas the challenge in genomics, proteomics and metabolomics has been to move from systems-level analysis to single cells, the challenge for electron microscopy has been to move from the single-cell level to a systems-level view of samples. vEM offers this opportunity.

The field is evolving rapidly, as more scientists realize the potential of vEM to contribute new insights into their research questions. This has attracted tool developers, in the form of microscopists, physicists, chemists, engineers and computational scientists, focused on improving the speed and throughput of vEM. Having originated in connectomics and neuroscience, the next grand challenge appears to be the mapping of the connectome of a single mouse brain. Rapid high-resolution image acquisition will also help to overcome the *n*-of-1 problem, allowing the analysis of multiple samples from a population to assess inter-organism variability. As we have already seen, technological advances focused on one challenge area (like the brain) quickly radiate out to drive discovery in other biological and clinical research areas.

A particular challenge lies in true multiscale imaging of tissues and organisms, to provide multidimensional views of samples with low-resolution and high-resolution information accessible from a single volume. The creation of multiscale digital twins will require parallel developments in light, X-ray and electron imaging, alongside improvements in sample preparation, multimodal probes and multiscale analysis and visualization. There are two crossover points in scales that currently block the creation of seamless image volumes from patient to protein. One is at the cryo-resin interface, at a sample thickness of between 1 μm and 200 μm, where 1 μm is the (somewhat generous) limit of continuous volume that can be imaged with cryo-ET to identify macromolecules in situ in cells, and 200 μm is the hard limit of vitrification, above which resin protocols effectively destroy or mask atomic structure. The second is at a sample thickness greater than 1 mm, above which diffusion limits the penetration of chemicals into the sample. Overcoming these blocks will require broad multidisciplinary collaboration between structural biologists, volume electron microscopists, light microscopists and pre-clinical and medical imaging scientists, with biologists, clinician scientists and the tool developers mentioned above.

The scale of this challenge requires investment into specialist facilities that gather a range of expertise and infrastructure. Deployment will then require roll out of new tools to specialist core facilities that will offer open access to a broad swathe of researchers, who will, in turn, depend on local facilities to perform fast effective sample preparation close to the end-user. Indeed, these local facilities are key in supporting the boom in vEM use, and are a source of valuable advice about experimental design and vEM modality selection for a specific biological question. However, the number of vEM experts is likely to become limiting as vEM technology is more widely adopted, a limitation already affecting the cryo-EM field, and it will be critical to develop and implement formal training programs, rather than the on-the-job training model currently used. Creation of a network of local, national and international open access vEM facilities will require a coordinated effort from the vEM community and funders, and will take time. In the meantime, vEM specialists and end-user researchers are encouraged to join the burgeoning community via the vEM community website or to contact their local electron microscopy facility for support and information.

If the effort to build a vEM infrastructure and community is successful, then we should see a move towards a new discipline of morphometrics, in which the number of samples imaged and analysed will provide statistically significant insights into the nanoscale spatial organization of organelles, cells, organs and systems. Crossing discipline boundaries, integration of spatial proteomics, transcriptomics and metabolomics data will add volume function to volume structure to drive a new era of functional morphometrics.

## Supplementary Material

Supplementary information is available for this paper at https://doi.org/10.1038/s43586-022-00131-9

Supplementary movie 1

Supplementary movie 2

## Data Availability

The two example data analysis workflows for neuron and mitochondria segmentation can be found at: https://github.com/kreshuklab/vem-primer-examples.Figure Legends

## Box 1 Experimental design

The choice of imaging modality for a research project should be carefully considered in view of the scientific question being asked. Key issues include the field of view required to image the entire structure of interest; the resolution required to observe the smallest feature of interest; conductivity of the sample after heavy metal staining and resin embedding, and hence tolerance of the sample to electron and/or ion beam exposure; variability of hardness of materials within the sample; and value of the sample, determining whether it can be imaged destructively (such as with tissue culture cells) or whether sample integrity should be maintained (in the case of human tissue).

Volume electron microscopy (vEM) approaches are slow and labour intensive, and so quality checks are crucial before the sample is subjected to extensive imaging and analysis. Thin section transmission electron microscopy (TEM) is appropriate to check quality of fixation and processing ahead of a vEM experiment. X-ray imaging of the resin-embedded specimens has also been used to assess overall quality for larger samples.

The low throughput of vEM limits the number of samples, and therefore the number of replicates, that can be analysed. This contributes to a difficulty in obtaining statistically robust data from biological systems with inherent variability in the population (for example, multiple cells). Quantification of objects (such as organelles and cells), lengths, surfaces, volumes, contacts between organelles and cell connectivity patterns inside a network would provide valuable information on the systems being studied, but the initial sampling strategy must mitigate the low throughput of vEM to reach significance. Often, vEM of defined regions must be used in parallel with more extensive quantitative analyses on multiple samples using 2D TEM and light microscopy.

A final issue relates to the significant challenges of processing, analysing and extracting features of interest from massive vEM image volumes. This requires specialist support, tools and software to extract meaning from the collected data. Preparations for data handling should start well before the first sample is imaged.

## Box 2 X-ray microscopy

X-ray microscopy, and in particular computed tomography, provides 3D information about the structure and morphology of intact samples ^283,284^. In many ways, the data that computed tomography provides are similar to that of volume electron microscopy (vEM) but at lower resolution. For example, elements with a higher atomic number tend to have both higher X-ray attenuation and higher electron scattering properties. Therefore, many of the same biological structures are visible with both techniques - especially when contrasted with heavy metals - and images can have a very similar appearance ^45,46,285–287^. Tomography works by taking 2D images of a sample from many different angles and combining these via a reconstruction algorithm to give a 3D tomogram. X-ray tomography can be done in the laboratory using laboratory-based X-ray systems or at large synchrotron facilities. In general, laboratory based systems can reach sub-micron resolutions with scan times in the range of a few hours to a few days ^44,283^, whereas synchrotrons can achieve resolutions of tens to hundreds of nanometres ^283,285,287^ (depending on the set-up chosen and the technique employed) with scan times in the range of seconds or minutes ^283,285^, although the highest resolution scans still take several hours ^287^. However, the benefits of synchrotron X-rays come at the cost of reduced accessibility, and care must be taken to avoid damage to the sample from high doses of X-rays ^288,289^. X-ray microscopy has many applications when combined with vEM. For example, computed tomography can be used to quickly screen samples to assess structural preservation ^173^ and stain penetration ^6^ prior to vEM. With its large field of view, it is also very useful as a targeting technique for correlative light and volume electron microscopy (vCLEM), as in conjunction with fast scan times it can efficiently produce 3D maps of samples to guide electron microscopy acquisition of regions of interest ^44–46,99,286^. Finally, 3D X-ray imaging can be used in its own right as the primary technique for morphological studies of samples, as demonstrated in recent studies tracing neurons in Drosophila and mouse brain ^285,287^.

## Glossary

Connectomics: An interdisciplinary field combining systems neuroscience, applied physics and computer science to map the physical and functional connections of the brain in a range of species.
Vibratome: An instrument for sectioning tissue that utilizes a vibrating blade.
Membrane tubulation: The process of membrane reorganization that produces tubular membrane invaginations or outward-facing tubular membrane projections.
Ultra-thin sections: Sections thin enough to allow transmission of an electron beam (usually 100 nm or less).
Diamond knife: A specialized tool used in electron microscopy where diamond is used to produce an extremely sharp and durable knife edge capable of cutting ultra-thin sections of tissue (down to about 30 nm).
Ultramicrotome: An instrument for preparing ultra-thin sections for electron microscopy.
Neural tracers: Fluorescent probes for imaging neuronal morphology.
Charging: A process that occurs in poorly conducting specimens in the scanning electron microscope as electrons are trapped and their excess negative charge causes surface potentials that distort the signal amplitude, focus and image geometry.
Isotropic voxels: 3D pixels with the same size in *x, y* and *z.*
Segmentation: The process of delineating objects within an image, usually with the aim of visualizing and/or analysing different object classes. Segmentation may be performed manually or using algorithms.
Noise: Unwanted alterations of the pixel intensities in an image caused by different stages of the imaging process (electron gun, signal detection, amplification).
Connectome: A map of the synaptic connections of the brain.
Choanocytes: Specialised cells found in sponges, contributing to feeding function via water circulation and filtration.
Tunnelling nanotubes: Extensions of the plasma membrane that form channels between cells whose proposed functions are to open communication channels between cells, for the exchange of organelles or other intracellular components.
Vitrification: Transformation of a substance into a non-crystalline, amorphous, glass-like solid. In cryoelectron microscopy (cryo-EM) this involves cryopreservation of materials without hexagonal ice crystal formation, to maintain cellular components in a near-native state.
Fiducial: An object added to the field of view of a microscope during imaging that can be used as a point of reference during sample preparation, imaging and image analysis.
Watershed Transform: An image processing technique that can be used for image segmentation by modelling the intensity of greyscale images as topographical peaks.
Classifier: An algorithm that categorises an observation based on quantifiable properties.
